# Endoscopic Ultrasound-Guided Radiofrequency Ablation (EUS-RFA): Are We Getting Evidence-Based Results? A Systematic Review According to the Levels of Evidence

**DOI:** 10.3390/medicina62071382

**Published:** 2026-07-17

**Authors:** Andrea Lisotti, Graziella Masciangelo, Matteo Tacelli, Stefano Francesco Crinò, Khanh Do-Cong Pham, Tawfik Khoury, Pietro Fusaroli, Bertrand Napoléon

**Affiliations:** 1 Gastroenterology Unit, Hospital of Imola, University of Bologna, 40026 Imola, Italy; masciangelo.lm@gmail.com (G.M.); pietro.fusaroli@unibo.it (P.F.); 2 IRCCS San Raffaele Scientific Institute, Vita-Salute San Raffaele University, 20132 Milan, Italy; tacelli.matteo@hsr.it; 3 Department of Medicine, University of Verona, 37134 Verona, Italy; stefanofrancesco.crino@aovr.veneto.it; 4 Department of Medicine, Haukeland University Hospital, N-5021 Bergen, Norway; phamkdc@gmail.com; 5 Department of Gastroenterology, Galilee Medical Center, Nahariya 22100, Israel; tawfikkhoury1@hotmail.com; 6 Azrieli Faculty of Medicine, Bar-Ilan University, 8 Henrietta Szold St, Safed 13115, Israel; 7 Endoscopy Unit, Hôpital Privé Jean Mermoz, 69008 Lyon, France; dr.napoleon@wanadoo.fr

**Keywords:** Endoscopic ultrasound, radiofrequency ablation, EUS-RFA, pancreatic neoplasms, pancreatic neuroendocrine tumors, intraductal papillary mucinous neoplasm, insulinoma, Oxford levels of evidence

## Abstract

*Background and Objectives:* Endoscopic ultrasound-guided radiofrequency ablation (EUS-RFA) is an emerging minimally invasive therapeutic option for pancreatic and selected extra-pancreatic lesions. However, its clinical adoption is limited by heterogeneous indications, non-standardized techniques, and variable quality of evidence. This systematic review assessed the published literature on EUS-RFA and classified available evidence according to the Oxford Centre for Evidence-Based Medicine levels of evidence. *Materials and Methods:* A systematic search was performed to identify peer-reviewed studies reporting clinical or translational data on EUS-RFA. Studies were grouped by indication, including pancreatic insulinoma, non-functioning pancreatic neuroendocrine neoplasms, branch-duct intraductal papillary mucinous neoplasms and other pancreatic cystic neoplasms, pancreatic ductal adenocarcinoma, pancreatic metastases, adrenal adenoma, and miscellaneous indications. Each study was categorized according to Oxford level of evidence based on study design. *Results*: Thirty-seven records were included in the final evidence map, comprising 36 clinical studies classifiable according to Oxford levels of evidence and one translational record not classifiable as clinical therapeutic evidence. Among the 36 clinically classifiable studies, one provided Level 1b evidence, consisting of a randomized trial evaluating EUS-guided celiac ganglion RFA for pancreatic cancer-related pain palliation, and three provided Level 2b evidence, including non-randomized comparative cohorts in pancreatic insulinoma and unresectable pancreatic ductal adenocarcinoma. Most clinically classifiable studies were Level 4 evidence (32/36), mainly uncontrolled prospective or retrospective cohorts and case series. One preclinical/translational study was not classifiable within clinical therapeutic evidence levels. Pancreatic insulinoma was the most evidence-supported tumor-ablation indication, with comparative data suggesting efficacy comparable to surgery and a more favorable safety profile. For non-functioning pancreatic neuroendocrine neoplasms, branch-duct IPMN, renal cell carcinoma pancreatic metastases, and adrenal adenomas, available data suggest feasibility and encouraging short-term outcomes but remain predominantly non-comparative. In pancreatic ductal adenocarcinoma, EUS-RFA remains investigational as an adjunct to systemic therapy. *Conclusions*: EUS-RFA is a promising therapeutic platform, but evidence remains highly indication-dependent and dominated by low-level observational studies. Standardized protocols, indication-specific outcomes, prospective registries, and comparative trials are needed.

## 1. Introduction

Endoscopic ultrasound-guided radiofrequency ablation (EUS-RFA) has emerged over the last decade as one of the most promising therapeutic applications of interventional EUS. By allowing real-time endosonographic targeting of solid or cystic lesions adjacent to the gastrointestinal lumen, EUS-RFA enables localized thermal ablation while avoiding surgical access [1,2]. This approach is particularly attractive for pancreatic and extra-pancreatic lesions located in anatomically complex regions, where surgery may be associated with substantial morbidity or may be precluded by patient frailty, comorbidities, advanced age, or refusal of resection.

The clinical use of EUS-RFA has progressively expanded across several indications. The most consolidated experience concerns pancreatic neuroendocrine neoplasms, particularly insulinomas and small non-functioning pancreatic neuroendocrine tumors, for which EUS-RFA has been proposed as a minimally invasive alternative to pancreatic surgery in selected patients. More recently, EUS-RFA has also been investigated for branch-duct intraductal papillary mucinous neoplasms, pancreatic ductal adenocarcinoma, pancreatic metastases, pancreatic cystic neoplasms, and selected extra-pancreatic targets such as left adrenal adenomas and hepatocellular carcinoma. However, these indications differ substantially in biological behavior, treatment intent, expected therapeutic endpoint, and acceptable risk threshold. For example, ablation of insulinoma aims primarily at control of hormonal hypersecretion, whereas ablation of non-functioning pancreatic neuroendocrine tumors or premalignant cystic lesions requires durable local disease control, and treatment of pancreatic ductal adenocarcinoma is generally investigational and adjunctive to systemic therapy [1,2,3].

Despite increasing clinical interest, the evidence supporting EUS-RFA remains heterogeneous and incompletely standardized. Published studies vary considerably in patient selection, lesion type and size, distance from the main pancreatic duct, ablation devices, generator settings, number and duration of applications, use of contrast-enhanced EUS, peri-procedural prophylaxis, post-procedural monitoring, imaging follow-up, and definitions of technical success, clinical success, radiological response, recurrence, and adverse events [3]. In addition, much of the available literature consists of retrospective cohorts, small prospective series, pilot studies, and case reports, with few comparative studies and no completed randomized trials for most tumor-ablation indications. As a consequence, the true position of EUS-RFA within the therapeutic algorithm of each disease remains uncertain, and the absence of standardized outcome reporting limits the comparability of studies and the development of evidence-based recommendations [4,5,6,7].

A rigorous appraisal of the existing literature is therefore required to distinguish indications supported by relatively mature evidence from those that remain exploratory. Classifying studies according to a recognized hierarchy of evidence may help clarify the robustness of current data, identify areas where EUS-RFA is approaching clinical implementation, and define priorities for future research. The Oxford Centre for Evidence-Based Medicine levels of evidence provide a practical framework for grading the methodological strength of therapeutic studies and for interpreting the relative weight of randomized, comparative observational, uncontrolled cohort, and case-based evidence.

The aim of this systematic review was to comprehensively assess the published literature on EUS-RFA across all reported clinical indications and to systematically classify the available evidence according to the Oxford levels of evidence. Secondary aims were to summarize the best available evidence for each indication, describe the main therapeutic outcomes and adverse events reported in the literature, and identify methodological gaps that should be addressed by future prospective studies and consensus-based standardization efforts.

## 2. Materials and Methods

### 2.1. Study Design

This study was a systematic review of the published literature on endoscopic ultrasound-guided radiofrequency ablation (EUS-RFA), with the specific aim of critically appraising the available evidence according to indication and study design, conducted according to Preferred Reporting Items for Systematic Reviews and Meta-Analyses (PRISMA) guidelines (https://www.prisma-statement.org/prisma-2020-statement, accessed on 25 April 2026). The methodology was developed in accordance with previous systematic reviews evaluating the levels of evidence in interventional and diagnostic EUS, and the reporting structure was aligned with the Preferred Reporting Items for Systematic Reviews and Meta-Analyses (PRISMA) framework. The review focused on clinical studies reporting the use of EUS-RFA for the treatment of gastrointestinal, pancreatic, hepatobiliary, or extra-luminal lesions accessible under EUS guidance. The primary objective was to classify the available literature by clinical indication and Oxford level of evidence. Secondary objectives were to summarize study design, population characteristics, procedural indications, technical feasibility, clinical outcomes, adverse events, and follow-up strategies. The review was not prospectively registered in PROSPERO or another registry, and no publicly available protocol was prepared before study initiation. Eligibility criteria, outcomes of interest, and evidence-classification rules were defined a priori by the authors before screening.

### 2.2. Literature Search Strategy

A systematic literature search was performed to identify peer-reviewed studies reporting original clinical data on endoscopic ultrasound-guided radiofrequency ablation (EUS-RFA). The search was initially performed on 29 May 2026 and subsequently updated during manuscript revision. The following databases were searched from inception to the final search date: PubMed/MEDLINE, Embase, Cochrane CENTRAL, Scopus, and Web of Science. In addition, ClinicalTrials.gov and the WHO International Clinical Trials Registry Platform were screened to identify ongoing or completed clinical studies. Search strings were adapted to the syntax of each database and are reported in Appendix A. Additional indication-specific searches were performed using terms related to pancreatic insulinoma, pancreatic neuroendocrine neoplasms, pancreatic ductal adenocarcinoma, intraductal papillary mucinous neoplasms, pancreatic cystic neoplasms, pancreatic metastases, adrenal adenoma, celiac ganglion ablation, and other extra-pancreatic EUS-RFA indications.

The reference lists of included articles and relevant reviews were manually screened to identify additional eligible studies. Only full-text articles published in peer-reviewed journals and written in English were considered eligible. Conference abstracts, editorials, letters without original clinical data, narrative reviews, systematic reviews, meta-analyses, animal studies, ex vivo studies, and technical reports without patient-level clinical outcomes were excluded.

### 2.3. Eligibility Criteria

Studies were eligible for inclusion if they met all of the following criteria: (1) evaluated EUS-guided radiofrequency ablation as the therapeutic intervention; (2) included human subjects; (3) reported original clinical data; (4) provided information on at least one clinically relevant outcome, including technical success, clinical success, radiological response, biochemical response, symptom control, adverse events, recurrence, progression, survival, or follow-up; and (5) were published in English in a peer-reviewed journal. The following study types were considered eligible: randomized controlled trials, prospective studies, retrospective studies, comparative cohort studies, and case series. Studies were excluded when: (1) animal or ex vivo studies; (2) technical notes without patient-level clinical outcomes; (3) case reports, narrative or systematic reviews, editorials, expert opinions, or commentaries without original data; (4) congress abstracts or conference proceedings not published as full-text peer-reviewed articles; (5) non-English-language publications; (6) studies evaluating non-EUS-guided radiofrequency ablation only; and (7) articles in which EUS-RFA was not the principal therapeutic intervention.

### 2.4. Study Selection

After completion of the search, titles and abstracts were screened for eligibility. Full-text articles were retrieved for all potentially relevant records. Two reviewers independently assessed each full-text article for inclusion. Disagreements were resolved by discussion and, when necessary, by consultation with a third reviewer. When multiple publications appeared to report overlapping populations from the same institution or study group, the most recent or most complete report was retained for the main analysis to avoid duplication of patient data. Earlier reports were considered only if they provided additional information not available in the later publication, such as longer follow-up, indication-specific outcomes, or detailed adverse event reporting.

### 2.5. Data Extraction

Data were extracted using a predefined standardized form. The following variables were collected from each included study: first author, year of publication, country, study design, study period, number of patients, number of lesions, target organ or disease indication, study design, and main outcomes reported.

### 2.6. Assessment of Levels of Evidence and Risk of Bias

Each included study was classified according to the Oxford Centre for Evidence-Based Medicine levels of evidence (https://www.cebm.ox.ac.uk/resources/levels-of-evidence/ocebm-levels-of-evidence, accessed on 25 April 2026). For therapeutic studies, systematic reviews of randomized controlled trials were classified as level 1 evidence; individual randomized controlled trials or high-quality prospective comparative studies were classified as level 2 evidence; non-randomized controlled cohort studies or comparative observational studies were classified as level 3 evidence; retrospective case series, uncontrolled prospective series, case–control studies were classified as level 4 evidence; and expert opinion or mechanism-based reasoning was classified as level 5 evidence. When meta-analyses included predominantly observational studies, the assigned level of evidence was interpreted according to the quality and design of the underlying primary studies rather than according to the publication type alone. For each indication, the highest available level of evidence and the predominant level of evidence were both recorded. Risk-of-bias and methodological quality assessment was performed independently by two reviewers (A.L. and G.M.). The assessment tool was selected according to study design. The revised Cochrane risk-of-bias tool for randomized trials (RoB 2) was used for randomized controlled trials. The Risk Of Bias In Non-randomized Studies of Interventions tool (ROBINS-I) was used for non-randomized comparative studies evaluating EUS-RFA against a comparator intervention or control group. Uncontrolled prospective or retrospective cohorts and case series were assessed using the Joanna Briggs Institute Critical Appraisal Checklist for Case Series. Disagreements were resolved by discussion and, when necessary, by consultation with a third reviewer (P.F.). Risk-of-bias and methodological quality judgments were not used to assign Oxford levels of evidence, which were based on study design. Instead, they were considered separately in the qualitative interpretation of the evidence for each indication. The certainty of evidence was assessed using a GRADE-informed approach at the indication level. GRADE domains were used to interpret the overall certainty of the body of evidence supporting each indication, together with publication bias. Certainty judgments were not used to reclassify Oxford levels of evidence. Characteristics, main findings, and Oxford level of evidence of the included studies are summarized in Supplementary Table S1. Risk of bias assessment of included studies is reported in Supplementary Table S2. The indication-level certainty assessment is reported in Supplementary Table S3. The method we handled the potential patients’ overlap among different studies is detailed in Supplementary Table S4. The technical EUS-RFA details and methodological study designs are described in Supplementary Table S5. Safety profiles and description of incidence of adverse events are reported in Supplementary Table S6. 

### 2.7. Quantitative Analysis

A quantitative meta-analysis was not planned and performed since the evidence base was characterized by substantial clinical and methodological heterogeneity across indications, lesion biology, treatment intent, devices and energy settings, number of ablation sessions, prophylactic measures, outcome definitions, follow-up duration, and study design.

### 2.8. Ethical Considerations

Because this study was based exclusively on previously published data, institutional review board approval and informed consent were not required. No individual patient-level unpublished data were used.

## 3. Results

### 3.1. Literature Search

**Figure 1:**
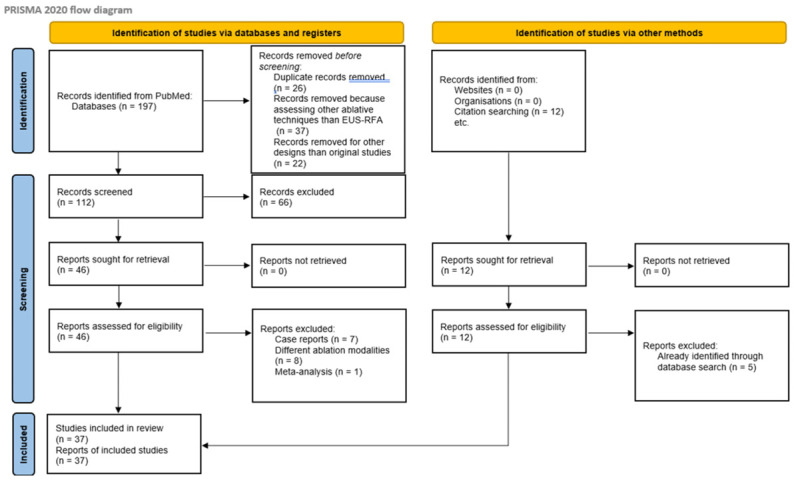
PRISMA flow-chart for literature search.

The systematic literature search identified 197 records through database searches. An additional 12 records were retrieved through citation searching. After removal of 26 duplicate records, 37 records were removed before screening because they assessed other ablative techniques than EUS-RFA, and 22 because they showed other study designs than original articles. Finally, 112 records underwent title and abstract screening. Of these, 66 records were excluded because they did not meet the eligibility criteria. Overall, 46 full-text reports were sought for retrieval, comprising 46 reports identified through database searching; of them, 7 were excluded because they were case reports and 8 because they evaluated ablation modalities other than EUS-guided radiofrequency ablation, and one was a meta-analysis. Moreover, 12 reports were identified through citation searching; of them, all were successfully retrieved and assessed for eligibility, and 5 of them were excluded since they were already included in the database search [8,9,10,11,12,13,14,15,16,17,18,19,20,21,22,23,24,25,26,27,28,29,30,31,32,33,34,35,36,37,38,39,40,41,42,43,44,45,46]. Finally, 37 studies were included in the systematic review [8,9,11,12,14,15,16,17,18,19,20,21,22,23,24,25,26,27,28,29,30,31,32,33,34,35,36,37,38,39,40,41,42,43,44,45,46] as shown in Figure 1

Overall, the final evidence map comprised 37 records. Of these, 36 were clinical studies classifiable according to Oxford levels of evidence and one was a translational/mechanistic record that was not classifiable as clinical therapeutic evidence. Among the 36 clinically classifiable studies, only one study provided Level 1b evidence and three studies provided Level 2b evidence. The vast majority of clinical studies consisted of Level 4 evidence (32/36), reflecting uncontrolled prospective or retrospective cohorts and case series. The translational record was retained separately to acknowledge mechanistic evidence on RFA-related immunomodulation, but it was not included in the clinical therapeutic evidence-level classification (Table 1). Rizzatti et al. reported a single prospective multicenter cohort of 60 patients, including 30 patients with insulinoma and 30 with non-functioning NF-pNENs. We describe subgroup outcomes in both sections for clinical clarity; however, the study was counted only once in the overall evidence map (in the NF-pNENs group) to avoid double-counting. Several reports originated from high-volume centers or collaborative groups with repeated publications on EUS-RFA. Potential overlap was identified for pancreatic cystic neoplasm/IPMN reports from the Marseille group, PDAC reports from the Chulalongkorn group, RCC pancreatic metastasis reports from the French/Marseille group, and safety-registry data from the French RAFPAN study. These reports were not combined quantitatively. For descriptive synthesis, the most specific or most mature report was preferentially used for indication-specific outcomes, whereas broader registry or earlier reports were used only when they provided complementary non-overlapping information.

Risk of bias assessment. The risk-of-bias and methodological quality assessment is summarized in Supplementary Table S2. Overall, the methodological quality of the evidence was limited by the predominance of uncontrolled observational studies, small sample sizes, heterogeneous inclusion criteria, variable follow-up duration, and non-standardized outcome definitions. The non-randomized comparative studies were affected mainly by potential residual confounding, selection bias, and imbalance in baseline clinical characteristics despite adjustment or matching strategies. Single-arm prospective and retrospective series generally showed acceptable reporting of technical feasibility and short-term safety, but were limited by the absence of comparator groups, incomplete reporting of consecutive inclusion, heterogeneous follow-up protocols, and limited assessment of long-term recurrence or disease-specific outcomes. The randomized trial on EUS-guided celiac ganglion radiofrequency ablation was considered separately because it addressed pain palliation rather than tumor ablation.

Certainty of evidence. The GRADE-informed certainty assessment is summarized in Supplementary Table S3. Overall, certainty was low or very low for most EUS-RFA indications. Pancreatic insulinoma represented the indication with the most mature evidence base, although certainty remained low for comparative effectiveness versus surgery because available data were mainly derived from non-randomized comparative evidence and small observational series. For non-functioning pancreatic neuroendocrine neoplasms and branch-duct IPMN or other pancreatic cystic neoplasms, certainty was very low to low, mainly because radiological or biochemical response was used as a surrogate for durable oncological control and because comparative long-term data were lacking. For pancreatic ductal adenocarcinoma, pancreatic metastases, mixed pancreatic neoplasm cohorts, and non-pancreatic tumor-ablation indications, certainty was very low because of uncontrolled or non-randomized study designs, small sample sizes, heterogeneous patient selection, variable concomitant therapies, and limited ability to separate local ablation effects from systemic disease biology or oncological treatment. The randomized trial of EUS-guided celiac ganglion radiofrequency ablation was considered separately because it supports pain-palliation outcomes rather than tumor-ablation efficacy.

Methodological heterogeneity. To characterize procedural heterogeneity, additional procedural variables, including lesion size, device or needle type, active-tip length, power or energy settings, number and duration of applications, number of treatment sessions, sedation strategy, antibiotic prophylaxis, pancreatitis prophylaxis, follow-up imaging modality and timing, and response definitions are summarized in Supplementary Table S5. Overall safety profile was reported in Supplementary Table S6.

### 3.2. Pancreatic Insulinoma

Pancreatic insulinoma represents the indication for EUS-guided radiofrequency ablation supported by the most robust clinical evidence. The highest-quality available study is a multicenter propensity-score matched comparison of EUS-RFA versus surgical resection in patients with sporadic pancreatic insulinoma. After matching, 89 patients were included in each treatment group [28]. Clinical efficacy was comparable between EUS-RFA and surgery, whereas EUS-RFA was associated with a substantially lower overall adverse-event rate, absence of severe adverse events, and significantly shorter hospital stay. Recurrence after EUS-RFA was reported in a minority of patients and was generally managed successfully with repeat EUS-RFA or surgery [28]. According to the Oxford Centre for Evidence-Based Medicine classification, this comparative non-randomized study represents level 2b evidence.

Prospective data further support the efficacy and safety of EUS-RFA in this setting. In the largest prospective international multicenter study available to date, 30 patients with insulinoma were treated with EUS-RFA [14]. Complete resolution of insulin hypersecretion-related symptoms was achieved in nearly all patients, with a low rate of adverse events. These findings are consistent with smaller retrospective series [13,15,24,26,35,46] and with pooled estimates from recent systematic reviews and meta-analyses, which report clinical success rates around 95% for EUS-RFA in insulinoma [5,6,7]. However, the overall evidence base remains limited by the predominance of observational studies, relatively small sample sizes, heterogeneous procedural protocols, and limited long-term follow-up.

Overall, EUS-RFA should be considered a highly effective minimally invasive treatment option for small sporadic pancreatic insulinomas, particularly in patients who are poor surgical candidates or who decline surgery. Current evidence suggests that EUS-RFA may provide symptom control comparable to surgery with lower procedural morbidity and shorter hospitalization. Nevertheless, surgery is still suggested as the reference standard by most guidelines; definitive positioning of EUS-RFA as first-line therapy requires confirmation from ongoing randomized comparative trials.

### 3.3. Pancreatic Non-Functioning Neuroendocrine Neoplasm

Non-functioning pancreatic neuroendocrine neoplasms (NF-pNENs) represent the second most investigated indication for pancreatic EUS-RFA, but the underlying evidence remains predominantly observational. The most robust prospective evidence is provided by the international multicenter study by Rizzatti et al. [14], which enrolled 30 patients with NF-pNENs measuring 15–25 mm, histologically confirmed as G1 or low-G2 lesions with Ki-67 ≤ 5%, without nodal or distant metastatic disease, and located more than 1 mm from the main pancreatic duct [14]. Among patients with long-term follow-up, complete radiological response was achieved in 22 of 25 patients (88%), while adverse events occurred in 5 of 30 NF-pNENs patients (16.7%). Only one severe adverse event was reported in the overall cohort, which included both functioning and non-functioning tumors [14]. Additional retrospective and imaging-based series support the feasibility of EUS-RFA in small localized NF-pNENs [9,12,34,39,41,43]. Platt et al. [9] described post-ablation imaging patterns in 17 patients with 18 pathology-proven localized NF-pNENs smaller than 3 cm treated with curative intent; lesion size, enhancement, and somatostatin-receptor PET uptake significantly decreased after ablation, with complete imaging response in 12 of 18 lesions (66.7%) and partial response in 5 of 18 lesions (27.8%). One post-ablation pancreatitis occurred. A recent systematic review and meta-analysis pooling EUS-guided ablation studies reported, for NF-pNENs treated with EUS-RFA, a pooled clinical success rate of 85% and a pooled adverse-event rate of 26%; however, the meta-analysis included mainly small observational cohorts and mixed EUS ablation techniques, and therefore does not overcome the intrinsic limitations of the primary evidence [9,10].

Overall, EUS-RFA for NF-pNENs appears technically feasible and potentially effective in carefully selected patients with small, localized, well-differentiated tumors, particularly when surgery is declined or considered high risk. Nevertheless, compared with insulinoma, the therapeutic endpoint is more demanding, because complete tumor eradication rather than hormonal symptom control is required. Moreover, long-term oncological outcomes, recurrence risk, optimal imaging criteria for complete response, and the oncological safety of replacing surveillance or surgery remain insufficiently defined. The best available evidence for this indication is therefore Oxford level 4, based on prospective and retrospective uncontrolled cohorts; no randomized trial or robust comparative study against surgery or active surveillance is currently available.

### 3.4. Pancreatic Ductal Adenocarcinoma

Pancreatic ductal adenocarcinoma represents an EUS-RFA indication evaluated mainly as an adjunct to systemic therapy in unresectable disease rather than as a curative local treatment. The best available clinical evidence is a prospective comparative study including patients with unresectable pancreatic ductal adenocarcinoma ≤ 4 cm treated with EUS-RFA plus systemic chemotherapy and matched controls receiving chemotherapy alone. After propensity-score matching and inverse probability weighting, EUS-RFA was associated with longer median survival compared with controls, with 12-month survival probabilities of 58% and 22%, respectively, and only mild abdominal pain reported in 10% of RFA procedures [16]. A previous observational open-label pilot study comparing EUS-RFA plus chemotherapy with chemotherapy alone showed significantly higher tumor necrosis and reduced narcotic requirements in the EUS-RFA group, although no significant difference in 6-month mortality was observed [23]. Additional single-arm data support technical feasibility and short-term safety. In a single-center historic cohort including 26 patients with locally advanced or metastatic pancreatic ductal adenocarcinoma, technical success was achieved in all cases, no major adverse events were reported, and local tumor reduction with post-treatment necrotic changes was observed among survivors at 6 months. However, overall survival remained poor, particularly in metastatic disease, and the absence of randomized trials, small sample sizes, heterogeneity in disease stage, concomitant chemotherapy, ablation protocols, and outcome definitions preclude definitive conclusions [22,31,33,38,46]. Overall, EUS-RFA for pancreatic ductal adenocarcinoma should currently be regarded as an investigational local cytoreductive strategy within multimodal treatment of selected patients with unresectable disease. The best available evidence is Oxford level 2b, derived from non-randomized comparative cohorts, whereas most supporting studies remain level 4 uncontrolled observational series.

### 3.5. Intraductal Papillary Mucinous Neoplasms

Branch-duct intraductal papillary mucinous neoplasms (BD-IPMNs) represent an emerging indication for EUS-RFA, particularly in patients with worrisome features or high-risk stigmata who are unsuitable for surgery or decline pancreatic resection. The best available evidence consists of two recent clinical studies specifically addressing BD-IPMN [8,11]. In a retrospective analysis of a prospectively maintained tertiary-center cohort, 50 patients with 58 BD-IPMN lesions underwent 62 EUS-RFA procedures over a long-term observation period. Technical success was achieved in all cases, with durable local control in almost all treated lesions and no pancreatic cancer arising from the ablated cyst during follow-up. Radiological response was frequent, although complete cyst disappearance occurred only in a minority of lesions. Adverse events were not negligible, occurring in approximately one quarter of procedures, but were mostly mild; post-procedural pancreatitis was the main clinically relevant complication, including one case of necrotizing pancreatitis [8].

Prospective data are available from a single-arm trial evaluating EUS-RFA in large BD-IPMNs. In this study, 25 patients with 30 large BD-IPMNs underwent 41 EUS-RFA sessions. Most cysts achieved at least 50% volume reduction during follow-up, and a subset showed near-complete volumetric response. Molecular response, assessed by disappearance of detectable KRAS/GNAS mutations in cyst fluid, was observed in most evaluable lesions, but remains exploratory and requires validation. Adverse events occurred in approximately 12% of procedures, with most classified as AGREE grade 3A [11]. Overall, these data suggest that EUS-RFA may provide local disease control and cyst-volume reduction in selected patients with BD-IPMN, especially when surgery is contraindicated or refused. However, the current evidence remains limited by non-randomized designs, small sample sizes, heterogeneous selection criteria, short-to-intermediate follow-up in prospective data, and the lack of validated oncological endpoints. Therefore, EUS-RFA for BD-IPMN should still be considered investigational and restricted to expert centers, preferably within prospective protocols or structured registries. The best available evidence is Oxford level 4.

### 3.6. Pancreatic Metastases

Pancreatic metastases represent a rare but clinically relevant indication for EUS-RFA, with the available evidence almost entirely limited to renal cell carcinoma metastases. The rationale for local ablation is particularly strong in this setting because pancreatic metastases from renal cell carcinoma may follow an indolent course, often occur after a long disease-free interval, and may be managed with focal therapy in selected oligometastatic patients in order to defer pancreatic surgery or systemic treatment. The best available evidence is a prospective, monocentric, single-arm pilot study including 12 patients with progressive pancreatic metastases from renal cell carcinoma [36]. Median lesion size was 17 mm, 21 pancreatic metastases were treated over 26 EUS-RFA procedures, and all procedures achieved technical success. After a median follow-up of 27.7 months, the 6- and 12-month focal control rates were 84.2% and 73.3%, respectively. No immediate adverse events occurred, but two clinically relevant delayed infectious adverse events were reported: one paraduodenal abscess in a patient receiving tyrosine kinase inhibitor therapy and one hepatic abscess in a patient with an indwelling biliary stent; both resolved with appropriate treatment. A retrospective observational study provided additional disease-specific evidence in 8 patients with 11 pancreatic renal cell carcinoma metastases treated with dedicated 18G or 19G RFA needles. Mean lesion size was 13.9 mm, technical success was 100%, and complete response was observed in 45.4% of lesions both at first CT follow-up and at 1 year, with partial response in an additional 27.3%. Three patients underwent repeated EUS-RFA. Adverse events occurred in three patients and included mild acute pancreatitis, abdominal pain, and pancreatic fistula with retro-gastric pseudocyst [20].

Overall, EUS-RFA for pancreatic metastases appears feasible and may achieve meaningful local disease control in highly selected patients with small pancreatic metastases from renal cell carcinoma. However, the evidence remains sparse, disease-specific, and non-comparative, with no randomized trials or robust comparisons against pancreatectomy, stereotactic radiotherapy, systemic therapy, or active surveillance. The role of EUS-RFA in pancreatic metastases from non-renal primaries is even less defined and is supported only by isolated cases or mixed small cohorts. Therefore, EUS-RFA should currently be considered an investigational focal treatment option for selected oligometastatic renal cell carcinoma patients, ideally after multidisciplinary tumor-board discussion and within expert centers. The best available evidence is Oxford level 4.

### 3.7. Adrenal Gland Adenoma

EUS-RFA has recently been investigated as a minimally invasive alternative to adrenalectomy for selected left-sided adrenal adenomas, particularly aldosterone-producing adenomas. The best available evidence is the FABULAS trial, a UK multicenter prospective proof-of-concept study evaluating EUS-guided RFA for left-sided aldosterone-producing adenomas [21]. In this study, 44 patients were screened and 28 participants were recruited; 35 ablations were performed, with 21 patients undergoing one ablation session and 7 requiring two sessions. All PET-CT-positive nodules were identified by EUS and successfully accessed for biopsy and ablation. The primary endpoint was procedural safety, defined by the occurrence of prespecified major hazards, including perforation, hemorrhage, or infarction of major organs within 24–48 h; none of these major hazards occurred. At 3 months, localized reduction in radiotracer uptake in the ablated adenoma was associated with complete or partial biochemical cure in 21 patients (75%), while complete or partial clinical cure of hypertension was achieved in 12 patients (43%). In four patients, disappearance of the adenoma on molecular imaging was associated with normotension off all antihypertensive treatment.

Additional evidence is available from a single-center prospective study of EUS-RFA for left adrenal tumors, which included 11 patients treated because of increasing tumor size or adrenal hormone excess. Technical success was achieved in all patients. After a median of two EUS-RFA sessions, complete response was reported in 73% and partial response in 27%. Safety outcomes were favorable, with only self-limiting mild abdominal pain in five patients and no moderate or severe adverse events [25].

Overall, EUS-RFA for adrenal adenomas appears technically feasible and safe when applied to left-sided lesions accessible from the stomach, with encouraging biochemical and clinical responses in aldosterone-producing adenomas. However, the evidence remains early and non-comparative. The available studies do not yet establish equivalence or superiority versus laparoscopic adrenalectomy, which remains the standard definitive treatment for unilateral primary aldosteronism in surgically fit patients. EUS-RFA may therefore be considered an investigational organ-sparing option for carefully selected patients, particularly those at increased surgical risk or those wishing to avoid adrenalectomy, and should be performed within expert multidisciplinary programs. The best available evidence is Oxford level 4, based on prospective uncontrolled studies; a comparative trial against surgery is still needed.

### 3.8. Other Indications

Additional indications for EUS-RFA have been reported in original studies, but the evidence is sparse, heterogeneous, and generally insufficient to support routine clinical use. Beyond BD-IPMN, pancreatic cystic neoplasms have been treated in small early series including mucinous cystadenoma, mucinous cystic neoplasms, and serous cystic neoplasms. In the initial human experience, EUS-RFA was applied to both pancreatic cystic neoplasms and neuroendocrine tumors, with complete cyst resolution in a minority of cystic lesions and partial reduction in others, without major adverse events [32,37,40,41,47,48]. Subsequent studies reported radiological responses in mucinous cystic lesions and IPMN, and a dedicated retrospective study in pancreatic microcystic serous cystic neoplasms showed partial radiological response in most patients, with only mild self-limiting abdominal pain. However, these data derive from very small uncontrolled cohorts, with heterogeneous cyst types, different response definitions, and limited oncological endpoints.

Solid pseudopapillary neoplasm represents another rare pancreatic indication. Published evidence is limited to very small experiences embedded within the broader literature on pancreatic neoplasms. A recent comprehensive review identified only two studies reporting EUS-RFA for small solid pseudopapillary neoplasms < 2 cm, with no recurrence during short follow-up. Given the intrinsic malignant potential of solid pseudopapillary neoplasms and the excellent long-term outcomes of surgical resection, EUS-RFA cannot currently be considered an alternative standard treatment, but only an investigational option in highly selected patients unfit for surgery or refusing resection.

A few studies also evaluated EUS-RFA outside the pancreas. For hepatocellular carcinoma, one case series reported EUS-RFA in five elderly patients with compensated cirrhosis and small HCCs, showing reduction in alpha-fetoprotein levels, radiological reduction after treatment, complete radiological response in lesions < 3 cm, and no procedural adverse events. Nevertheless, this evidence remains preliminary, and percutaneous or surgical ablation remains the established standard for most ablatable HCCs. Finally, EUS-guided celiac ganglion or celiac plexus RFA has been assessed as a palliative intervention for pancreatic cancer-related pain. This indication is mechanistically distinct from tumor ablation, because the target is neural tissue rather than the neoplastic lesion. In a single-blind randomized trial, celiac ganglion RFA was compared with EUS-guided celiac plexus neurolysis for pain palliation in locally advanced or metastatic pancreatic cancer. Although this represents higher-level evidence than most tumor-ablation indications, it should be categorized separately from oncological EUS-RFA because the therapeutic endpoint is pain control rather than tumor response or disease modification [42].

Overall, miscellaneous indications for EUS-RFA are supported mainly by case series, pilot studies, or highly selected retrospective cohorts. Except for celiac ganglion RFA for pain palliation, for which randomized comparative evidence is available, the best available evidence for these miscellaneous tumor indications is Oxford level 4. These applications should therefore be regarded as investigational and should be restricted to expert centers, multidisciplinary decision-making, and preferably prospective registries or formal clinical trials.

## 4. Discussion

This systematic review shows that EUS-guided radiofrequency ablation is a rapidly expanding therapeutic EUS technique with encouraging clinical results across several pancreatic and extra-pancreatic indications, but with an evidence base that remains uneven, indication-dependent, and largely non-standardized. The main finding is that EUS-RFA cannot be interpreted as a single intervention supported by a uniform body of evidence [4,5,6,7]; rather, its evidentiary maturity varies substantially according to disease setting. Insulinoma is currently the indication with the strongest support, including comparative non-randomized evidence against surgery, whereas non-functioning pancreatic neuroendocrine neoplasms, branch-duct intraductal papillary mucinous neoplasms, pancreatic metastases, adrenal adenomas, and miscellaneous indications are supported mainly by prospective or retrospective uncontrolled cohorts. Pancreatic ductal adenocarcinoma occupies an intermediate but still investigational position, with emerging comparative data suggesting a potential adjunctive role in selected patients with unresectable disease. Overall, our results support the original hypothesis that EUS-RFA is an emerging and clinically promising intervention but that robust evidence and procedural standardization remain insufficient for broad, indication-agnostic implementation [3].

The most consistent evidence was identified for pancreatic insulinoma. In this setting, the therapeutic endpoint is clinically direct and measurable: resolution of hypoglycemic symptoms and normalization of biochemical hyperinsulinism. This may partly explain why outcomes appear more reproducible than for other indications. The available comparative evidence suggests that EUS-RFA may achieve symptom control comparable to surgery while reducing morbidity, severe adverse events, and length of hospitalization. These findings are clinically relevant because insulinomas are often small, benign or low-grade tumors, and the morbidity of pancreatic surgery may be disproportionate to the biological aggressiveness of the disease in selected patients. However, the absence of randomized evidence prevents definitive replacement of surgery as the standard reference treatment. At present, EUS-RFA appears most defensible as an alternative in patients at increased surgical risk, those refusing surgery, or those in whom parenchyma-sparing treatment is strongly preferred after multidisciplinary assessment [28].

For non-functioning pancreatic neuroendocrine neoplasms, the interpretation is more complex [14]. Unlike insulinoma, the objective is not merely symptom control but durable oncological eradication or long-term disease control. The available prospective data show high rates of complete radiological response in carefully selected small, localized, well-differentiated lesions. Nevertheless, radiological disappearance or absence of contrast enhancement cannot yet be considered fully equivalent to histological cure. This distinction is central to the clinical positioning of EUS-RFA in non-functioning lesions, where active surveillance and surgery are both accepted options depending on tumor size, grade, growth, patient age, comorbidity, and preferences. The current evidence supports feasibility and short-term efficacy, but long-term recurrence, metastatic risk, and optimal post-ablation surveillance remain insufficiently defined. Therefore, EUS-RFA for non-functioning pancreatic neuroendocrine neoplasms should currently be regarded as a promising organ-sparing strategy for selected patients rather than a standardized replacement for surgery or surveillance.

Branch-duct IPMN represents another attractive but still investigational application [8,11]. The rationale is strong: many patients with worrisome features are elderly or comorbid, pancreatic surgery carries significant morbidity, and a substantial proportion of resected IPMNs do not harbor high-grade dysplasia or invasive cancer. EUS-RFA may therefore offer an organ-preserving option for patients unsuitable for resection or unwilling to undergo surgery. Recent studies show encouraging local control, cyst-volume reduction, and exploratory molecular response. However, IPMN is a field-defect disease involving the entire gland, and local ablation of a target cyst cannot eliminate the risk of metachronous pancreatic cancer elsewhere in the pancreas. This explains why local control and pancreas-wide disease control must be interpreted separately. Moreover, the clinically meaningful endpoint in IPMN should not be cyst shrinkage alone, but prevention of progression to high-grade dysplasia or invasive carcinoma over sufficiently long follow-up. Current data are promising but not yet adequate to define EUS-RFA as an alternative standard to surgery or surveillance.

In pancreatic ductal adenocarcinoma, EUS-RFA should be interpreted through a different conceptual framework [16,22]. The clinically relevant endpoints in PDAC are difficult to attribute specifically to EUS-RFA. Overall survival, local control, pain palliation, performance status, and quality of life may all be influenced by systemic therapy, tumor biology, disease stage, biliary or duodenal obstruction, nutritional status, and patient selection. Therefore, apparent survival or local-control signals cannot be considered evidence of independent oncological benefit from EUS-RFA in the absence of randomized or adequately controlled comparative data. Similarly, improvement in pain or performance status may reflect multimodal treatment, supportive care, or selection of patients with more favorable disease biology. Accordingly, EUS-RFA for PDAC should currently be regarded as investigational. The available data support feasibility and preliminary safety in selected patients treated by experienced interventional endosonographers, but they do not establish EUS-RFA as a standard adjunct to chemotherapy or as a validated strategy for downstaging, local control, or survival improvement. Future prospective studies should include clearly defined PDAC subgroups, standardized ablation protocols, predefined systemic treatment pathways, and clinically meaningful endpoints, including local progression-free survival, overall survival, pain-control metrics, quality of life, adverse events, and treatment-related delays in systemic therapy.

The evidence for pancreatic metastases is narrower but biologically coherent, being almost entirely focused on renal cell carcinoma [20,36]. This is an important distinction because pancreatic metastases from renal cell carcinoma often behave indolently, may remain oligometastatic, and can be considered for focal therapy. EUS-RFA achieved meaningful local control in small prospective and retrospective series, suggesting that it may defer pancreatectomy or systemic treatment in selected patients. However, this evidence cannot be generalized to pancreatic metastases from other primaries, which may have different biology, growth kinetics, systemic treatment options, and prognosis. Future studies should therefore avoid grouping all pancreatic metastases together and should instead analyze renal cell carcinoma separately from other metastatic diseases.

The extra-pancreatic evidence, particularly for left adrenal adenomas, expands the conceptual boundaries of interventional EUS-RFA. The FABULAS trial and other prospective data suggest that EUS-RFA may be technically feasible and safe for left-sided adrenal lesions, with encouraging biochemical and clinical responses in aldosterone-producing adenomas [21]. This is an important development because it indicates that EUS-RFA may evolve beyond pancreatic disease into a broader platform for minimally invasive ablation of lesions adjacent to the gastrointestinal tract. Nevertheless, laparoscopic adrenalectomy remains the reference treatment for unilateral primary aldosteronism in surgically fit patients, and comparative studies are required before EUS-RFA can be incorporated into standard endocrine practice.

A major cross-cutting finding of this review is the lack of standardization [3]. Across studies, there was substantial variability in eligibility criteria, lesion-size thresholds, distance from the main pancreatic duct, device type, active-tip length, power settings, duration of energy delivery, number of applications, use of contrast-enhanced EUS, prophylaxis against pancreatitis or infection, admission policy, and follow-up schedule. Outcome definitions were similarly heterogeneous. Technical success was usually reported consistently, but clinical success, complete response, local control, recurrence, and adverse events were defined differently across indications and studies. This heterogeneity limits evidence synthesis and precludes reliable comparison between studies. Standardized definitions are urgently needed, and they should be indication-specific. For example, clinical success in insulinoma should require durable symptom resolution and biochemical control; in non-functioning pancreatic neuroendocrine neoplasms it should include radiological and functional imaging response; in IPMN it should distinguish cyst response from pancreas-wide progression; and in pancreatic ductal adenocarcinoma it should include oncological endpoints such as local control, progression-free survival, overall survival, pain control, and interaction with systemic therapy.

A key finding of this review is that favorable short-term outcomes should not be interpreted as definitive evidence of durable disease control. Across indications, several distinct endpoints were variably reported, including technical success, clinical or biochemical response, radiological response, local control, recurrence, progression-free survival, and disease-specific survival. These outcomes are not interchangeable. Technical success only confirms successful deployment of the RFA probe and energy delivery into the target lesion. Clinical response, such as resolution of hypoglycemia in insulinoma, reflects symptom control but does not necessarily prove complete eradication of neoplastic tissue. Radiological response, including reduction or disappearance of enhancement, may suggest local treatment effect but remains a surrogate endpoint. Conversely, durable local control, absence of recurrence, progression-free survival, and disease-specific survival require longer follow-up and, ideally, comparator groups. Therefore, especially for non-functioning pancreatic neuroendocrine neoplasms, branch-duct IPMN or other pancreatic cystic neoplasms, pancreatic ductal adenocarcinoma, and pancreatic metastases, the currently available evidence should be interpreted as supporting feasibility and short-term response rather than established long-term oncological safety.

This review has several strengths. First, it provides a comprehensive indication-based appraisal of the EUS-RFA literature rather than focusing on a single disease entity. Second, by classifying each study according to Oxford levels of evidence, it offers a transparent framework to distinguish relatively mature indications from exploratory applications. Third, the review highlights not only efficacy and safety outcomes, but also the methodological limitations that currently prevent widespread standardization. This approach is particularly useful in a rapidly evolving field where technical feasibility often precedes high-quality comparative evidence. Several limitations should also be acknowledged. The conclusions are constrained by the quality of the underlying literature. Most included studies were retrospective cohorts, prospective single-arm studies, small case series, and only a minority included comparator groups. Sample sizes were often small, follow-up was variable, and overlapping patient populations may have been present in sequential publications from high-volume centers. The absence of prospective registration is an additional methodological limitation. Publication bias is likely, because successful early experiences and novel indications are more likely to be reported than negative or complicated cases. A further limitation is the possibility of partial overlap among reports from high-volume centers and collaborative networks. We attempted to minimize this issue by identifying potentially overlapping cohorts and avoiding aggregation of patient numbers or outcomes across these reports. However, because individual patient-level identifiers were unavailable, residual overlap cannot be completely excluded. Moreover, this review was restricted to full-text articles published in English. Therefore, language bias cannot be excluded, and relevant studies published in other languages may have been missed, particularly for an emerging technique such as EUS-RFA that may be reported in regional or non-English journals. Finally, because the review spans multiple diseases with different natural histories and therapeutic goals, direct comparison of outcomes across indications should be avoided.

The clinical implications of these findings are substantial. EUS-RFA appears closest to clinical integration for small pancreatic insulinomas, where the balance between efficacy and reduced morbidity is most favorable. For non-functioning pancreatic neuroendocrine neoplasms and BD-IPMN, EUS-RFA should be considered an organ-preserving investigational option in carefully selected patients after multidisciplinary discussion. For pancreatic ductal adenocarcinoma, it should be studied as an adjunct to systemic therapy rather than as a stand-alone treatment. For renal cell carcinoma pancreatic metastases and adrenal adenomas, early results justify further prospective disease-specific research. Across all indications, the implementation of EUS-RFA should be limited to expert centers with interventional EUS expertise, access to pancreatic surgery, radiology, oncology, and structured follow-up.

Future research should prioritize prospective multicenter registries using standardized data elements and indication-specific outcome definitions. Randomized or well-designed comparative studies are particularly needed for insulinoma, non-functioning pancreatic neuroendocrine neoplasms, and locally advanced pancreatic ductal adenocarcinoma. For BD-IPMN, long-term studies should evaluate progression to high-grade dysplasia or invasive cancer rather than relying only on cyst-volume reduction. For all pancreatic indications, future protocols should clarify optimal power settings, active-tip selection, number and duration of applications, safety margins from the main pancreatic duct, prophylaxis strategies, post-procedural monitoring, and imaging follow-up. Harmonization of reporting will be essential before evidence-based guidelines and consensus recommendations can be developed.

## 5. Conclusions

In conclusion, EUS-RFA is a promising minimally invasive therapeutic platform with expanding applications in pancreatic and selected extra-pancreatic diseases. However, the strength of evidence remains highly indication-dependent. The only Level-1 evidence was focused on pancreatic cancer pain palliation through EUS-RFA of the celiac ganglia. Among tumor-ablation indications, pancreatic insulinoma currently represents the most mature clinical application; however, available evidence remains mainly observational, and long-term recurrence-free outcomes require further confirmation. For non-functioning pancreatic neuroendocrine neoplasms, pancreatic cystic neoplasms including BD-IPMN, pancreatic ductal adenocarcinoma, pancreatic metastases, adrenal lesions, and other emerging indications, current evidence supports technical feasibility and preliminary local response but does not establish durable oncological efficacy or long-term safety. Therefore, outside highly selected indications, EUS-RFA should still be regarded as investigational and should preferably be performed in expert centers, after multidisciplinary evaluation, and within prospective clinical protocols or structured registries.

## Figures and Tables

**Table 1:** Summary of levels of evidence according to the EUS-RFA indication.

Indication/Study Subject	LE 1	LE 2	LE 3	LE 4	Not Applicable	Total
Pancreatic insulinoma	–	1	–	5	–	6
Non-functioning or mixed pancreatic neuroendocrine neoplasms	–	–	–	6	–	6
Branch-duct IPMN and pancreatic cystic neoplasms	–	–	–	6	–	6
Pancreatic ductal adenocarcinoma	–	2	–	5	–	7
Mixed pancreatic neoplasms/safety and feasibility cohorts	–	–	–	5	–	5
Pancreatic metastases from renal cell carcinoma	–	–	–	2	–	2
Non-pancreatic tumor ablation indications	–	–	–	3	–	3
Celiac ganglion RFA for pancreatic cancer pain palliation	1	–	–	–	–	1
Preclinical/translational pancreatic cancer study	–	–	–	–	1	1
Total	1	3	0	32	1	37

*Abbreviations*: LE, level of evidence; EUS-RFA, endoscopic ultrasound-guided radiofrequency ablation; IPMN, intraductal papillary mucinous neoplasm; RFA, radiofrequency ablation. Footnote: Oxford levels of evidence were assigned only to clinical studies reporting therapeutic or palliative clinical outcomes. The final evidence map included 37 records, of which 36 were clinically classifiable according to Oxford levels of evidence and one translational/mechanistic record was not classifiable as clinical therapeutic evidence. The non-classifiable translational record was retained separately and was not included in the denominator for Oxford-classifiable clinical evidence. Isolated case reports, systematic reviews, and meta-analyses were excluded from the included-study set.

## Data Availability

No new data were created or analyzed in this study. Data sharing is not applicable to this article..

## Supplementary Materials

The following supporting information can be downloaded at https://www.mdpi.com/article/10.3390/medicina62071382/s1: PRISMA checklist and PRISMA flowchart are available as supplementary materials.Supplementary Table S1: Characteristics, main findings, and Oxford level of evidence of the included studies; Supplementary Table 2: Risk of bias assessment of included studies; Supplementary Table S3: Certainty of evidence according to GRADE classification; Supplementary Table S4: Handling of potential study overlap; Supplementary Table S5: EUS-RFA technique and study designs; Supplementary Table S6: Incidence of adverse events.

## Abbreviations

The following abbreviations are used in this manuscript:

EUS-RFA	Endoscopic ultrasound radiofrequency ablation
IPMN	Intraductal papillary mucinous neoplasm
PDAC	Pancreatic ductal adenocarcinoma
NEN	Neuroendocrine neoplasm
NF-pNENs	Non-functioning pancreatic NENs

## Appendix A. Database-Specific Search Strategies

The search strategy was developed to identify studies evaluating endoscopic ultrasound-guided radiofrequency ablation across pancreatic, gastrointestinal, hepatobiliary, and extra-pancreatic indications. Searches were performed from database inception to the final search date, without publication-date restrictions. The core strategy combined terms related to endoscopic ultra-sound with terms related to radiofrequency ablation, and database-specific syntax was adapted according to each database. In PubMed/MEDLINE, the following search string was used: (“endoscopic ultrasound”[Title/Abstract] OR “endoscopic ultraso-nography”[Title/Abstract] OR “endosonography”[Title/Abstract] OR “EUS”[Title/Abstract]) AND (“radiofrequency abla-tion”[Title/Abstract] OR “radio-frequency ablation”[Title/Abstract] OR “RFA”[Title/Abstract] OR “thermal abla-tion”[Title/Abstract]). Additional indication-specific PubMed/MEDLINE searches combined the same EUS-RFA terms with disease-specific terms, including “insulinoma,” “pancreatic neuroendocrine tumor,” “pancreatic neuroendocrine tumour,” “pancreatic neuroendocrine neoplasm,” “PanNET,” “pNET,” “non-functioning pancreatic neuroendocrine tumor,” “pancreatic cancer,” “pancreatic ductal adenocarcinoma,” “PDAC,” “pancreatic cystic neoplasm,” “pancreatic cystic lesion,” “intraductal papillary mucinous neoplasm,” “IPMN,” “branch-duct IPMN,” “mucinous cystic neoplasm,” “serous cystic neoplasm,” “pancreatic metastasis,” “pancreatic metastases,” “renal cell carcinoma,” “RCC,” “adrenal adenoma,” “aldosterone-producing adenoma,” “hepatocellular carcinoma,” “HCC,” “celiac ganglion,” and “celiac plexus.”

In Embase, the core search strategy was: (‘endoscopic ultrasound’:ti,ab,kw OR ‘endoscopic ultrasonography’:ti,ab,kw OR en-dosonography:ti,ab,kw OR EUS:ti,ab,kw) AND (‘radiofrequency ablation’:ti,ab,kw OR ‘radio-frequency ablation’:ti,ab,kw OR RFA:ti,ab,kw OR ‘thermal ablation’:ti,ab,kw). This was supplemented by indication-specific searches combining the same EUS-RFA terms with the following disease-specific terms in title, abstract, or keyword fields: insulinoma, pancreatic neuro-endocrine tumor, pancreatic neuroendocrine tumour, pancreatic neuroendocrine neoplasm, PanNET, pNET, non-functioning pancreatic neuroendocrine tumor, pancreatic cancer, pancreatic ductal adenocarcinoma, PDAC, pancreatic cystic neoplasm, intraductal papillary mucinous neoplasm, IPMN, pancreatic metastasis, pancreatic metastases, renal cell carcinoma, RCC, adrenal adenoma, aldosterone-producing adenoma, hepatocellular carcinoma, HCC, celiac ganglion, and celiac plexus.

In Cochrane CENTRAL, the following search strategy was used: (“endoscopic ultrasound” OR “endoscopic ultrasonography” OR endosonography OR EUS) AND (“radiofrequency ablation” OR “radio-frequency ablation” OR RFA OR “thermal abla-tion”). Additional searches combined these terms with indication-specific keywords, including insulinoma, pancreatic neuro-endocrine tumor, pancreatic neuroendocrine neoplasm, PanNET, pNET, pancreatic cancer, pancreatic ductal adenocarcinoma, PDAC, pancreatic cystic neoplasm, intraductal papillary mucinous neoplasm, IPMN, pancreatic metastasis, renal cell carci-noma, RCC, adrenal adenoma, aldosterone-producing adenoma, hepatocellular carcinoma, HCC, celiac ganglion, and celiac plexus.

In Scopus, the search was performed using title, abstract, and keyword fields as follows: TITLE-ABS-KEY (“endoscopic ultra-sound” OR “endoscopic ultrasonography” OR endosonography OR EUS) AND TITLE-ABS-KEY (“radiofrequency ablation” OR “radio-frequency ablation” OR RFA OR “thermal ablation”). Additional indication-specific searches combined the same EUS-RFA terms with TITLE-ABS-KEY terms for insulinoma, pancreatic neuroendocrine tumor, pancreatic neuroendocrine tumour, pancreatic neuroendocrine neoplasm, PanNET, pNET, pancreatic cancer, pancreatic ductal adenocarcinoma, PDAC, pancreatic cystic neoplasm, intraductal papillary mucinous neoplasm, IPMN, pancreatic metastasis, pancreatic metastases, renal cell carcinoma, RCC, adrenal adenoma, aldosterone-producing adenoma, hepatocellular carcinoma, HCC, celiac ganglion, and celiac plexus.

In Web of Science, the following topic search was used: TS = (“endoscopic ultrasound” OR “endoscopic ultrasonography” OR endosonography OR EUS) AND TS = (“radiofrequency ablation” OR “radio-frequency ablation” OR RFA OR “thermal abla-tion”). Additional topic searches combined the same EUS-RFA terms with indication-specific terms including insulinoma, pancreatic neuroendocrine tumor, pancreatic neuroendocrine tumour, pancreatic neuroendocrine neoplasm, PanNET, pNET, pancreatic cancer, pancreatic ductal adenocarcinoma, PDAC, pancreatic cystic neoplasm, intraductal papillary mucinous neo-plasm, IPMN, pancreatic metastasis, pancreatic metastases, renal cell carcinoma, RCC, adrenal adenoma, aldoste-rone-producing adenoma, hepatocellular carcinoma, HCC, celiac ganglion, and celiac plexus.

ClinicalTrials.gov was searched using the terms “endoscopic ultrasound radiofrequency ablation,” “EUS-RFA,” “radiofre-quency ablation,” and the broader combination “endoscopic ultrasound” OR “EUS” AND “radiofrequency ablation” OR “RFA.” Disease-specific terms were also used, including pancreatic, adrenal, hepatocellular carcinoma, celiac ganglion, and celiac plexus. The WHO International Clinical Trials Registry Platform was searched using the terms “endoscopic ultrasound” OR “EUS” OR “endosonography” combined with “radiofrequency ablation” OR “RFA” OR “thermal ablation.” Finally, the reference lists of included articles and relevant reviews were manually screened to identify additional eligible studies not re-trieved through the electronic database searches.

## References

[1] Coluccio C., Cappetta S., Romagnoli G., Di Giorgio V., Giuffrida P., Fabbri S., Fabbri C., Binda C. (2025). Endoscopic-Ultrasound-Guided Radiofrequency Ablation for Pancreatic Tumors. J. Clin. Med..

[2] Masciangelo G., Campana D., Ricci C., Andrini E., Rakichevikj E., Fusaroli P., Lisotti A. (2025). Endoscopic Ultrasound-Guided Locoregional Treatments for Pancreatic Neuroendocrine Neoplasms. Curr. Oncol..

[3] Lisotti A., Tacelli M., Crinò S.F., Masciangelo G., Fusaroli P., Barthet M., Thosani N., Conti Bellocchi M.C., Leblanc S., Khoury T. (2026). Methodology Assessment of Endoscopic Ultrasound Radiofrequency Ablation (EUS-RFA) for Pancreatic Neoplasms: Results from an International Survey. Dig. Endosc..

[4] Armellini E., Facciorusso A., Crinò S.F. (2023). Efficacy and Safety of Endoscopic Ultrasound-Guided Radiofrequency Ablation for Pancreatic Neuroendocrine Tumors: A Systematic Review and Metanalysis. Medicina.

[5] Garg R., Mohammed A., Singh A., Harnegie M.P., Rustagi T., Stevens T., Chahal P. (2022). EUS-guided radiofrequency and ethanol ablation for pancreatic neuroendocrine tumors: A systematic review and meta-analysis. Endosc. Ultrasound.

[6] Khoury T., Sbeit W., Fusaroli P., Campana D., Brighi N., Napoleon B., Lisotti A. (2024). Safety and efficacy of endoscopic ultrasound-guided radiofrequency ablation for pancreatic neuroendocrine neoplasms: Systematic review and meta-analysis. Dig. Endosc..

[7] Kim H.J., Seo D.W., Hassanuddin A., Kim S.H., Chae H.J., Jang J.W., Park D.H., Lee S.S., Lee S.K., Kim M.H. (2012). EUS-guided radiofrequency ablation of the porcine pancreas. Gastrointest. Endosc..

[8] Barras J., Lorenzo D., Gasmi M., Gonzalez J.M., Barthet M. (2026). Endoscopic ultrasound-guided radiofrequency ablation for intraductal papillary mucinous neoplasms with worrisome features: Long-term outcomes in non-surgical patients (with video). Gastrointest. Endosc..

[9] Platt S., Gonda T., Asare B., Melamud K., Chetlur P., Huang C. (2026). Imaging Features of Pancreatic Neuroendocrine Tumors Following Radiofrequency Ablation: Early Experience. J. Comput. Assist. Tomogr..

[10] Matsumoto K., Fujii Y., Uchida D., Takeuchi Y., Mitsuhashi T., Otsuka M. (2026). Clinical efficacy and safety of endoscopic ultrasound-guided ablation therapies for pancreatic neuroendocrine tumors: A systematic review and meta-analysis. Ther. Adv. Gastroenterol..

[11] Krishna S.G., Park E., Rath J., Shah Z., Abdelbaki A., Culp S., Hawa F., Jones D., Chen W., Lee P. (2026). Endoscopic ultrasound-guided radiofrequency ablation for large branch-duct intraductal papillary mucinous neoplasms: Safety and efficacy trial. Endosc. Int. Open.

[12] Ardengh A.O., Micelli-Neto O., Venco F.E., Panizza P., Blasbalg R., Kemp R., Sebastião Dos Santos J., Ardengh J.C. (2025). Tumor enhancement by magnetic resonance imaging after endoscopic ultrasound-guided radiofrequency ablation for small pancreatic neuroendocrine tumors. VideoGIE.

[13] Adeniran O., Chittajallu V., Almusabeh H., Abdullahi S., Qureshi H., Adekolu A., Singh S. (2025). Successful Endoscopic Ultrasound-Guided Radiofrequency Ablation for Symptomatic Insulinoma in a High-Risk Elderly Patient. ACG Case Rep. J..

[14] Rizzatti G., Napoléon B., Caillol F., Crinó S.F., de Nucci G., Pham K.D., Giovannini M., Leblanc S., Della Torre S., Palazzo L. (2025). Endoscopic ultrasound-guided radiofrequency ablation for treatment of pancreatic neuroendocrine tumors: Multicenter prospective study. Endosc. Int. Open.

[15] Kovacevic B., Vilmann P., Brink L., Klose M.C., Sonne M.P., Knigge U., Andreassen M. (2026). EUS-Guided Radiofrequency Ablation as a Minimally Invasive Treatment for Insulinomas—A Single-Center Experience. J. Clin. Endocrinol. Metab..

[16] Kongkam P., Tantitanawat K., Kerr S., Lopimpisuth C., Tiankanon K., Angsuwatcharakon P., Ridtitid W., Mekaroonkamol P., Teeyapun N., Tanasanvimon S. (2025). One-year survival rate of unresectable pancreatic cancer size 4 cm or smaller treated with or without EUS-radiofrequency ablation. Gastrointest. Endosc..

[17] Okasha H.H., Altonbary A.Y., Ragab K., Ghoneem E., Tag-Adeen M., Abdellatef A., Naguib M.S., Miutescu B., Gadour E. (2025). Endoscopic ultrasound-guided radiofrequency ablation (EUS-RFA) and endoscopic ultrasound-guided ethanol ablation (EUS-EA) of pancreatic neuroendocrine tumors and adenocarcinoma: A prospective multicenter study. Gastroenterol. Rev..

[18] Harwani Y., Butala S., Shukla V., Patel A., Jani S.D. (2025). Treatment of Hepatocellular Carcinoma Using Endoscopic Ultrasound-guided Radiofrequency Ablation: A Case Series. DEN Open.

[19] Goduguchinta V., Ebrahim M., Patel R., Randhawa N., Khalyfa A., Inamullah M., Desai R., Ayub K. (2025). Safety and Efficacy of Radiofrequency Ablation in Management of Various Pancreatic Neoplasms. J. Clin. Med..

[20] Stouvenot M., Koch S., Frontzcak A., D’Engremont C., Boinette A., Doussot A., Maurina T., Vuitton L. (2025). Effectiveness and safety of endoscopic ultrasound-guided radiofrequency ablation for pancreatic metastases of renal cell carcinoma. Endosc. Int. Open.

[21] Argentesi G., Wu X., Ney A., Goodchild E., Laycock K., Lee Y.N., Senanayake R., MacFarlane J., Ng E., Kearney J. (2025). Endoscopic, ultrasound-guided, radiofrequency ablation of aldosterone-producing adenomas (FABULAS): A UK, multicentre, prospective, proof-of-concept trial. Lancet.

[22] Robles-Medranda C., Del Valle R., Puga-Tejada M., Arevalo-Mora M., Cunto D., Egas-Izquierdo M., Estrada-Guevara L., Bunces-Orellana O., Moreno-Zambrano D., Alcivar-Vasquez J. (2024). Assessing EUS-guided radiofrequency ablation in unresectable pancreatic ductal adenocarcinoma: A single-center historic cohort study. Gastrointest. Endosc..

[23] Kongkam P., Tiankanon K., Seo D.W., Luangsukrerk T., Sriuranpong V., Nantavithya C., Jantarattana T., Cañones A., Kerr S.J., Tantitanawat K. (2023). EUS-guided radiofrequency ablation plus chemotherapy versus chemotherapy alone for pancreatic cancer (ERAP): An observational open-label pilot study. Endosc. Ultrasound.

[24] Debraine Z., Borbath I., Deprez P., Bosly F., Maiter D., Furnica R.M. (2024). Long-term clinical and radiological outcomes of endoscopic ultrasound-guided radiofrequency ablation of benign insulinomas. Clin. Endocrinol..

[25] Cho S.H., Kim D.H., Seo D.W., Yoo S.K., Oh D., Song T.J., Lee S.K. (2023). Expanded indication for EUS-guided radiofrequency ablation: Management of adrenal tumors. Gastrointest. Endosc..

[26] Borrelli de Andreis F., Boškoski I., Mascagni P., Schepis T., Bianchi A., Schinzari G., Annicchiarico B.E., Quero G., Tortora G., Alfieri S. (2023). Safety and efficacy of endoscopic ultrasound-guided radiofrequency ablation for pancreatic insulinoma: A single-center experience. Pancreatology.

[27] Napoléon B., Lisotti A., Caillol F., Gasmi M., Ah-Soune P., Belle A., Charachon A., Cholet F., Eyraud P.Y., Grandval P. (2023). Risk factors for EUS-guided radiofrequency ablation adverse events in patients with pancreatic neoplasms: A large national French study (RAFPAN study). Gastrointest. Endosc..

[28] Crinò S.F., Napoleon B., Facciorusso A., Lakhtakia S., Borbath I., Caillol F., Do-Cong Pham K., Rizzatti G., Forti E., Palazzo L. (2023). Endoscopic Ultrasound-guided Radiofrequency Ablation Versus Surgical Resection for Treatment of Pancreatic Insulinoma. Clin. Gastroenterol. Hepatol..

[29] Faraoni E.Y., O’Brien B.J., Strickland L.N., Osborn B.K., Mota V., Chaney J., Atkins C.L., Cen P., Rowe J., Cardenas J. (2023). Radiofrequency Ablation Remodels the Tumor Microenvironment and Promotes Neutrophil-Mediated Abscopal Immunomodulation in Pancreatic Cancer. Cancer Immunol. Res..

[30] Figueiredo Ferreira M., Garces-Duran R., Eisendrath P., Devière J., Deprez P., Monino L., Van Laethem J.L., Borbath I. (2022). EUS-guided radiofrequency ablation of pancreatic/peripancreatic tumors and oligometastatic disease: An observational prospective multicenter study. Endosc. Int. Open.

[31] Thosani N., Cen P., Rowe J., Guha S., Bailey-Lundberg J.M., Bhakta D., Patil P., Wray C.J. (2022). Endoscopic ultrasound-guided radiofrequency ablation (EUS-RFA) for advanced pancreatic and periampullary adenocarcinoma. Sci. Rep..

[32] Younis F., Ben-Ami Shor D., Lubezky N., Geva R., Osher E., Shibolet O., Phillips A., Scapa E. (2022). Endoscopic ultrasound-guided radiofrequency ablation of premalignant pancreatic-cystic neoplasms and neuroendocrine tumors: Prospective study. Eur. J. Gastroenterol. Hepatol..

[33] Oh D., Seo D.W., Song T.J., Park D.H., Lee S.K., Kim M.H. (2022). Clinical outcomes of EUS-guided radiofrequency ablation for unresectable pancreatic cancer: A prospective observational study. Endosc. Ultrasound.

[34] Marx M., Godat S., Caillol F., Poizat F., Ratone J.P., Pesenti C., Schoepfer A., Hoibian S., Dahel Y., Giovannini M. (2022). Management of non-functional pancreatic neuroendocrine tumors by endoscopic ultrasound-guided radiofrequency ablation: Retrospective study in two tertiary centers. Dig. Endosc..

[35] Marx M., Trosic-Ivanisevic T., Caillol F., Demartines N., Schoepfer A., Pesenti C., Ratone J.P., Robert M., Giovannini M., Godat S. (2022). EUS-guided radiofrequency ablation for pancreatic insulinoma: Experience in 2 tertiary centers. Gastrointest. Endosc..

[36] Chanez B., Caillol F., Ratone J.P., Pesenti C., Rochigneux P., Pignot G., Thomassin J., Brunelle S., Walz J., Salem N. (2021). Endoscopic Ultrasound-Guided Radiofrequency Ablation as an Future Alternative to Pancreatectomy for Pancreatic Metastases from Renal Cell Carcinoma: A Prospective Study. Cancers.

[37] Barthet M., Giovannini M., Gasmi M., Lesavre N., Boustière C., Napoleon B., LaQuiere A., Koch S., Vanbiervliet G., Gonzalez J.M. (2021). Long-term outcome after EUS-guided radiofrequency ablation: Prospective results in pancreatic neuroendocrine tumors and pancreatic cystic neoplasms. Endosc. Int. Open.

[38] Wang J., Wang Y., Zhao Y., Wu X., Zhang M., Hou W., Chen Q., Cheng B. (2021). Endoscopic ultrasound-guided radiofrequency ablation of unresectable pancreatic cancer with low ablation power and multiple applications: A preliminary study of 11 patients. Ann. Palliat. Med..

[39] de Nucci G., Imperatore N., Mandelli E.D., di Nuovo F., d’Urbano C., Manes G. (2020). Endoscopic ultrasound-guided radiofrequency ablation of pancreatic neuroendocrine tumors: A case series. Endosc. Int. Open.

[40] Oh D., Ko S.W., Seo D.W., Hong S.M., Kim J.H., Song T.J., Park D.H., Lee S.K., Kim M.H. (2021). Endoscopic ultrasound-guided radiofrequency ablation of pancreatic microcystic serous cystic neoplasms: A retrospective study. Endoscopy.

[41] Barthet M., Giovannini M., Lesavre N., Boustiere C., Napoleon B., Koch S., Gasmi M., Vanbiervliet G., Gonzalez J.M. (2019). Endoscopic ultrasound-guided radiofrequency ablation for pancreatic neuroendocrine tumors and pancreatic cystic neoplasms: A prospective multicenter study. Endoscopy.

[42] Bang J.Y., Sutton B., Hawes R.H., Varadarajulu S. (2019). EUS-guided celiac ganglion radiofrequency ablation versus celiac plexus neurolysis for palliation of pain in pancreatic cancer: A randomized controlled trial (with videos). Gastrointest. Endosc..

[43] Choi J.H., Seo D.W., Song T.J., Park D.H., Lee S.S., Lee S.K., Kim M.H. (2018). Endoscopic ultrasound-guided radiofrequency ablation for management of benign solid pancreatic tumors. Endoscopy.

[44] Crinò S.F., D’Onofrio M., Bernardoni L., Frulloni L., Iannelli M., Malleo G., Paiella S., Larghi A., Gabbrielli A. (2018). EUS-guided Radiofrequency Ablation (EUS-RFA) of Solid Pancreatic Neoplasm Using an 18-gauge Needle Electrode: Feasibility, Safety, and Technical Success. J. Gastrointest. Liver Dis..

[45] Lakhtakia S., Ramchandani M., Galasso D., Gupta R., Venugopal S., Kalpala R., Reddy D.N. (2016). EUS-guided radiofrequency ablation for management of pancreatic insulinoma by using a novel needle electrode (with videos). Gastrointest. Endosc..

[46] Song T.J., Seo D.W., Lakhtakia S., Reddy N., Oh D.W., Park D.H., Lee S.S., Lee S.K., Kim M.H. (2016). Initial experience of EUS-guided radiofrequency ablation of unresectable pancreatic cancer. Gastrointest. Endosc..

[47] Khoury T., Farraj M., Sbeit W., Lisotti A., Napoléon B. (2025). Solid Pseudopapillary Neoplasm of the Pancreas: A Comprehensive Review Focusing on the Role of Endoscopic Ultrasound-Guided Radiofrequency Ablation as an Alternative Treatment. Cancers.

[48] Coupier A., Khoury T., Gincul R., Fumex F., Lisotti A., Leblanc S., Napoléon B. (2023). Endoscopic ultrasound-guided radiofrequency ablation for solid pseudopapillary neoplasm of the pancreas. Endoscopy.

