# Supplementary material for: Endoscopic Ultrasound-Guided Radiofrequency Ablation (EUS-RFA): Are We Getting Evidence-Based Results? A Systematic Review According to the Levels of Evidence
Source: Medicina (Kaunas). 2026 Jul 17;62(7):1382. doi: 10.3390/medicina62071382 (PMC13414305; doi:10.3390/medicina62071382)
Supplement: Supplementary file 1 [file medicina-62-01382-s001.zip › medicina-4378340-supplementary_R1.pdf]

*Type of the Paper (Systematic review)*

# Endoscopic ultrasound-guided radiofrequency ablation (EUS-RFA): are we getting evidence-based? A systematic review according to the levels of evidence.

**Andrea Lisotti [1,*], Graziella Masciangelo [1], Matteo Tacelli [2], Stefano Francesco Crinò [3], Khanh Do-Cong Pham [4], Tawfik Khoury [5], Pietro Fusaroli [1], and Bertrand Napoléon [6]**

**Supplementary Table S1.** Characteristics, main findings, and Oxford level of evidence of the included studies

| Reference | Title | PMID | Main findings | Oxford level of evidence |
|---|---|---|---|---|
| Barras et al. *Gastrointest Endosc*, 2026 [8] | EUS-guided radiofrequency ablation for intraductal papillary mucinous neoplasms with worrisome features: long-term outcomes in non-surgical patients | 41903818 | In 50 non-surgical patients with 58 BD-IPMN lesions, EUS-RFA achieved 100% technical success and durable local control in almost all treated lesions. No treated lesion progressed to cancer, although adverse events occurred in approximately one quarter of procedures. | Level 4 |
| Platt et al. *J Comput Assist Tomogr*, 2026 [9] | Imaging Features of Pancreatic Neuroendocrine Tumors Following Radiofrequency Ablation: Early Experience | 41656678 | In 17 patients with 18 localized non-functioning panNETs, post-ablation imaging showed marked reductions in lesion size, enhancement, and somatostatin-receptor PET uptake. Complete imaging response was observed in two thirds of lesions. | Level 4 |
| Krishna et al. *Endosc Int Open*, 2026 [11] | Endoscopic ultrasound-guided radiofrequency ablation for large branch-duct intraductal papillary mucinous neoplasms: Safety and efficacy trial | 41584732 | In a prospective single-arm trial of 25 patients with 30 large BD-IPMNs, most lesions achieved ≥50% cyst-volume reduction, and a subset achieved near-complete response. Molecular response based on KRAS/GNAS disappearance was promising but exploratory. | Level 4 |
| Ardengh et al. *VideoGIE*, 2025 [12] | Tumor enhancement by magnetic resonance imaging after endoscopic ultrasound-guided radiofrequency ablation for small pancreatic neuroendocrine tumors | 41467165 | Four small pancreatic neuroendocrine tumors were treated with EUS-RFA, with MRI demonstrating complete destruction of the nodules. The study supports the feasibility of MRI for post-ablation response assessment. | Level 4 |
| Rizzatti et al. *Endosc Int Open*, 2025 [14] | Endoscopic ultrasound-guided radiofrequency ablation for treatment of pancreatic neuroendocrine tumors: Multicenter prospective study | 41142260 | In a prospective multicenter study of 60 patients with functional and non-functional PanNETs, EUS-RFA achieved symptom resolution in nearly all insulinoma patients and complete radiological response in most evaluable NF-PanNETs. Adverse | Level 4 |

| Reference | Title | PMID | Main findings | Oxford level of evidence |
|---|---|---|---|---|
| | | | events were infrequent, with only one severe event in the whole cohort. | |
| Kovacevic et al. *J Clin Endocrinol Metab*, 2026 [15] | EUS-Guided Radiofrequency Ablation as a Minimally Invasive Treatment for Insulinomas—A Single-Center Experience | 41120112 | In 17 patients with insulinoma, EUS-RFA achieved clinical success in all cases, although approximately one third required a second session. Adverse events were mild or moderate. | Level 4 |
| Kongkam et al. *Gastrointest Endosc*, 2025 [16] | One-year survival rate of unresectable pancreatic cancer size 4 cm or smaller treated with or without EUS-radiofrequency ablation | 40680897 | In a comparative cohort of unresectable pancreatic cancer ≤4 cm, EUS-RFA plus chemotherapy was associated with improved median and 12-month survival compared with chemotherapy alone after adjustment. Reported RFA-related adverse events were mild. | Level 2b |
| Okasha et al. *Prz Gastroenterol*, 2025 [17] | Endoscopic ultrasound-guided radiofrequency ablation and ethanol ablation of pancreatic neuroendocrine tumors and adenocarcinoma: a prospective multicenter study | 40620311 | This prospective multicenter study evaluated EUS-RFA and EUS-guided ethanol ablation in pancreatic neuroendocrine tumors and adenocarcinoma, supporting feasibility across mixed indications. Interpretation is limited by mixed interventions and heterogeneous disease types. | Level 4 |
| Harwani et al. *DEN Open*, 2025 [18] | Treatment of Hepatocellular Carcinoma Using Endoscopic Ultrasound-guided Radiofrequency Ablation: A Case Series | 40599977 | Five elderly patients with small hepatocellular carcinomas underwent EUS-RFA, with radiological response and no procedure-related adverse events. Evidence remains preliminary and limited to a small case series. | Level 4 |
| Goduguchinta et al. *J Clin Med*, 2025 [19] | Safety and Efficacy of Radiofrequency Ablation in Management of Various Pancreatic Neoplasms | 40507720 | This small mixed-indication pancreatic neoplasm series suggested that EUS-RFA is technically feasible and may provide local response in selected unresectable or high-risk patients. The study is limited by heterogeneity and absence of a comparator. | Level 4 |
| Stouvenot et al. *Endosc Int Open*, 2025 [20] | Effectiveness and safety of endoscopic ultrasound-guided radiofrequency ablation for pancreatic metastases of renal cell carcinoma | 40376026 | In 8 patients with 11 renal cell carcinoma pancreatic metastases, technical success was 100%, with complete or partial response in most lesions. Adverse events included pancreatitis, abdominal pain, and pancreatic fistula/pseudocyst. | Level 4 |
| Argentesi et al. *Lancet*, 2025 [21] | Endoscopic, ultrasound-guided, radiofrequency ablation of aldosterone-producing adenomas: FABULAS trial | 39929216 | In this prospective proof-of-concept trial of left-sided aldosterone-producing adenomas, all PET-positive nodules were accessed and ablated without major early safety hazards. Biochemical cure or | Level 4 |

| Reference | Title | PMID | Main findings | Oxford level of evidence |
|---|---|---|---|---|
| | | | improvement was observed in most patients, but no surgical comparator was included. | |
| Robles-Medranda et al. *Gastrointest Endosc*, 2024 [22] | Assessing EUS-guided radiofrequency ablation in unresectable pancreatic ductal adenocarcinoma: a single-center historic cohort study | 38518978 | In 26 patients with unresectable or metastatic PDAC, EUS-RFA was technically successful in all cases and no major adverse events were reported. Local tumor reduction and necrotic changes were observed, but survival remained poor, especially in metastatic disease. | Level 4 |
| Kongkam et al. *Endosc Ultrasound*, 2023 [23] | EUS-guided radiofrequency ablation plus chemotherapy versus chemotherapy alone for pancreatic cancer: ERAP observational open-label pilot study | 37969163 | This observational pilot comparison suggested that EUS-RFA plus chemotherapy increased tumor necrosis and reduced narcotic requirements compared with chemotherapy alone. No significant improvement in 6-month mortality was demonstrated. | Level 2b |
| Debraine et al. *Clin Endocrinol*, 2024 [24] | Long-term clinical and radiological outcomes of endoscopic ultrasound-guided radiofrequency ablation of benign insulinomas | 37859570 | This long-term insulinoma series reported durable clinical and radiological outcomes after EUS-RFA, supporting its role as a minimally invasive alternative in selected patients. Evidence remains uncontrolled. | Level 4 |
| Cho et al. *Gastrointest Endosc*, 2023 [25] | Expanded indication for EUS-guided radiofrequency ablation: management of adrenal tumors | 37356635 | In 11 patients with left adrenal tumors, EUS-RFA achieved technical success in all cases and complete or partial response in all patients after a median of two sessions. Only mild self-limited abdominal pain was reported. | Level 4 |
| Borrelli de Andreis et al. *Pancreatology*, 2023 [26] | Safety and efficacy of endoscopic ultrasound-guided radiofrequency ablation for pancreatic insulinoma: a single-center experience | 37236853 | This single-center insulinoma series showed high rates of clinical success after EUS-RFA with acceptable safety. The findings support feasibility but remain limited by retrospective design and small sample size. | Level 4 |
| Napoléon et al. *Gastrointest Endosc*, 2023 [27] | Risk factors for EUS-guided radiofrequency ablation adverse events in patients with pancreatic neoplasms: RAFPAN study | 37059368 | This large retrospective national study evaluated adverse events after EUS-RFA for pancreatic neoplasms and identified risk factors for complications. It provides important safety data but no comparative efficacy assessment. | Level 4 |
| Crinò et al. *Clin Gastroenterol Hepatol*, 2023 [28] | Endoscopic Ultrasound-guided Radiofrequency Ablation Versus Surgical Resection for Treatment of Pancreatic Insulinoma | 36871765 | In a propensity-score matched comparison of 89 EUS-RFA patients and 89 surgical patients, EUS-RFA achieved similar clinical efficacy with fewer overall and severe adverse events and shorter | Level 2b |

| Reference | Title | PMID | Main findings | Oxford level of evidence |
|---|---|---|---|---|
| | | | hospitalization. This is the strongest comparative evidence for insulinoma. | |
| Faraoni et al. *Cancer Immunol Res*, 2023 [29] | Radiofrequency Ablation Remodels the Tumor Microenvironment and Promotes Neutrophil-Mediated Abscopal Immunomodulation in Pancreatic Cancer | 36367967 | Translational/mechanistic record; not classifiable as clinical therapeutic evidence. | Not applicable |
| Figueiredo Ferreira et al. *Endosc Int Open*, 2022 [30] | EUS-guided radiofrequency ablation of pancreatic/peripancreatic tumors and oligometastatic disease: observational prospective multicenter study | 36262511 | This prospective multicenter observational study supported the feasibility of EUS-RFA across pancreatic/peripancreatic tumors and oligometastatic disease. The mixed population and absence of a comparator limit indication-specific conclusions. | Level 4 |
| Thosani et al. *Sci Rep*, 2022 [31] | Endoscopic ultrasound-guided radiofrequency ablation for advanced pancreatic and periampullary adenocarcinoma | 36192558 | This prospective series showed that EUS-RFA can be performed in advanced pancreatic and periampullary adenocarcinoma, supporting feasibility in malignant disease. Efficacy conclusions remain limited by the uncontrolled design. | Level 4 |
| Younis et al. *Eur J Gastroenterol Hepatol*, 2022 [32] | Endoscopic ultrasound-guided radiofrequency ablation of premalignant pancreatic-cystic neoplasms and neuroendocrine tumors: prospective study | 36170679 | In a small prospective study of pancreatic cystic neoplasms and neuroendocrine tumors, EUS-RFA was feasible and associated with radiological response in selected lesions. Evidence is limited by small sample size and mixed indications. | Level 4 |
| Oh et al. *Endosc Ultrasound*, 2022 [33] | Clinical outcomes of EUS-guided radiofrequency ablation for unresectable pancreatic cancer: prospective observational study | 35083978 | This prospective observational study of unresectable pancreatic cancer supported technical feasibility and short-term safety of EUS-RFA. Oncological benefit remains uncertain due to lack of a comparator. | Level 4 |
| Marx et al. *Dig Endosc*, 2022 [34] | Management of non-functional pancreatic neuroendocrine tumors by EUS-guided radiofrequency ablation: retrospective study in two tertiary centers | 34963025 | This bicentric retrospective study suggested that EUS-RFA may achieve radiological response in selected small non-functioning pNETs. The study supports feasibility but lacks long-term comparative oncological outcomes. | Level 4 |
| Marx et al. *Gastrointest Endosc*, 2022 [35] | EUS-guided radiofrequency ablation for pancreatic insulinoma: experience in two tertiary centers | 34902374 | In a small two-center insulinoma experience, EUS-RFA achieved symptom control in most patients with an acceptable safety profile. Evidence remains limited by sample size and retrospective design. | Level 4 |
| Chanez et al. *Cancers*, 2021 [36] | EUS-guided radiofrequency ablation as an alternative to pancreatectomy for pancreatic metasta- | 34771431 | In 12 patients with renal cell carcinoma pancreatic metastases, all 26 EUS-RFA procedures were technically successful. Focal control rates were encour- | Level 4 |

| Reference | Title | PMID | Main findings | Oxford level of evidence |
|---|---|---|---|---|
| | ses from renal cell carcinoma: prospective study | | aging at 6 and 12 months, although delayed infectious complications occurred in two patients. | |
| Barthet et al. *Endosc Int Open*, 2021 [37] | Long-term outcome after EUS-guided radiofrequency ablation: prospective results in pancreatic neuroendocrine tumors and pancreatic cystic neoplasms | 34447860 | This long-term follow-up study of pancreatic neuroendocrine tumors and cystic neoplasms supported sustained response after EUS-RFA in selected cases. The mixed population and lack of comparator limit definitive conclusions. | Level 4 |
| Wang et al. *Ann Palliat Med*, 2021 [38] | EUS-guided radiofrequency ablation of unresectable pancreatic cancer with low ablation power and multiple applications: preliminary study | 33440967 | This preliminary study suggested that low-power, multiple-application EUS-RFA is feasible in unresectable pancreatic cancer. Evidence remains early and uncontrolled. | Level 4 |
| de Nucci et al. *Endosc Int Open*, 2020 [39] | EUS-guided radiofrequency ablation of pancreatic neuroendocrine tumors: a case series | 33269307 | This prospective case series reported technical feasibility and clinical/radiological response after EUS-RFA of pancreatic neuroendocrine tumors. The study contributed early clinical evidence but was uncontrolled and small. | Level 4 |
| Oh et al. *Endoscopy*, 2021 [40] | EUS-guided radiofrequency ablation of pancreatic microcystic serous cystic neoplasms: retrospective study | 33063298 | In patients with pancreatic microcystic serous cystic neoplasms, EUS-RFA produced partial radiological response in most treated lesions. Only mild self-limiting adverse events were reported. | Level 4 |
| Barthet et al. *Endoscopy*, 2019 [41] | EUS-guided radiofrequency ablation for pancreatic neuroendocrine tumors and pancreatic cystic neoplasms: prospective multicenter study | 30669161 | This prospective multicenter study provided early evidence that EUS-RFA is feasible in pancreatic neuroendocrine tumors and cystic neoplasms, with encouraging response rates. The absence of a comparator and mixed indications limit inference. | Level 4 |
| Bang et al. *Gastrointest Endosc*, 2019 [42] | EUS-guided celiac ganglion radiofrequency ablation versus celiac plexus neurolysis for palliation of pain in pancreatic cancer: randomized controlled trial | 30120957 | This randomized trial compared EUS-guided celiac ganglion RFA with celiac plexus neurolysis for pancreatic cancer pain palliation. It provides the highest-level evidence in the dataset, although the indication is pain palliation rather than tumor ablation. | Level 1b |
| Choi et al. *Endoscopy*, 2018 [43] | EUS-guided radiofrequency ablation for management of benign solid pancreatic tumors | 29727904 | This prospective single-center study showed that EUS-RFA was feasible for benign solid pancreatic tumors, including neuroendocrine lesions. Findings were encouraging but based on an uncontrolled cohort. | Level 4 |
| Crinò et al. *J Gastrointestin Liver Dis*, 2018 [44] | EUS-RFA of solid pancreatic neoplasm using an 18-gauge needle | 29557417 | This early consecutive case series demonstrated the technical feasibility and short-term safety of EUS-RFA using an 18-gauge needle electrode for solid | Level 4 |

| Reference | Title | PMID | Main findings | Oxford level of evidence |
|---|---|---|---|---|
| | electrode: feasibility, safety, and technical success | | pancreatic neoplasms. It primarily provided feasibility evidence. | |
| Lakhtakia et al. *Gastrointest Endosc*, 2016 [45] | EUS-guided radiofrequency ablation for management of pancreatic insulinoma by using a novel needle electrode | 26394384 | This early human insulinoma series showed that EUS-RFA using a dedicated needle electrode could achieve symptom control in a small number of patients. It represented one of the first clinical applications of EUS-RFA for insulinoma. | Level 4 |
| Song et al. *Gastrointest Endosc*, 2016 [46] | Initial experience of EUS-guided radiofrequency ablation of unresectable pancreatic cancer | 26344883 | This initial clinical experience demonstrated the feasibility of EUS-RFA in unresectable pancreatic cancer. The study was limited by very small sample size and lack of comparative outcome data. | Level 4 |

**Abbreviations:** BD-IPMN, branch-duct intraductal papillary mucinous neoplasm; EUS-RFA, endoscopic ultrasound-guided radiofrequency ablation; PanNET, pancreatic neuroendocrine tumor; PDAC, pancreatic ductal adenocarcinoma; RCC, renal cell carcinoma.

**Supplementary Table S2**. Risk of bias assessment of included studies.

| Reference (PMID) | Indication target | Study design | Tool | Main methodological concerns | Overall judgment |
|---|---|---|---|---|---|
| Barras et al. Gastrointest Endosc, 2026 [8] PMID: 41903818 | BD-IPMN / pancreatic cystic neoplasms | Retrospective analysis of a prospectively maintained single-center cohort | JBI Case Series | Selected non-surgical population; no comparator; potential center/operator selection bias; heterogeneous WF/HRS profile; response mainly radiological/local-control based. | Moderate methodological concerns |
| Platt et al. J Comput Assist Tomogr, 2026 [9] PMID: 41656678 | Localized non-functioning pancreatic neuroendocrine tumors | Retrospective imaging-based case series | JBI Case Series | Internal database selection; small sample; no comparator; imaging response endpoints; variable imaging modalities and timing; limited oncological follow-up. | Moderate methodological concerns |
| Krishna et al. Endosc Int Open, 2026 [11] PMID: 41584732 | Large BD-IPMN | Prospective single-arm trial | JBI Case Series | No comparator; small sample; selected patients declining or unfit for surgery; intermediate follow-up; molecular response exploratory; response analyzed per lesion while adverse events per session. | Moderate methodological concerns |

| Reference (PMID) | Indication target | Study design | Tool | Main methodological concerns | Overall judgment |
|---|---|---|---|---|---|
| Ardengh et al. VideoGIE, 2025 [12] PMID: 41467165 | Small pancreatic neuro-endocrine tumors | Very small case series with imaging follow-up | JBI Case Series | Only four tumors; no comparator; short and heterogeneous follow-up; imaging-focused endpoint; limited external validity. | High methodological concerns |
| Rizzatti et al. Endosc Int Open, 2025 [14] PMID: 41142260 | Functional and non-functioning pancreatic neuro-endocrine tumors | Prospective multicenter single-arm cohort | JBI Case Series | No comparator; selected small well-differentiated lesions; limited follow-up; subgroup outcomes by tumor function; potential overlap with high-volume European centers. | Moderate methodological concerns |
| Kovacevic et al. J Clin Endocrinol Metab, 2026 [15] PMID: 41120112 | Pancreatic insulinoma | Single-center observational case series | JBI Case Series | No comparator; small sample; likely referral/operator selection; repeat sessions required in some patients; limited long-term recurrence assessment. | Moderate methodological concerns |
| Kongkam et al. Gastrointest Endosc, 2025 [16] PMID: 40680897 | Unresectable pancreatic cancer <=4 cm | Non-randomized comparative cohort | ROBINS-I | Non-random treatment allocation; potential residual confounding despite adjustment; survival affected by chemotherapy, stage, performance status, and selection; open-label design. | Serious risk of bias |
| Okasha et al. Prz Gastroenterol, 2025 [17] PMID: 40620311 | Mixed pancreatic neuro-endocrine tumors and adenocarcinoma | Prospective multicenter mixed-intervention study | JBI Case Series | Mixed indications and ablation modalities; small subgroups; no comparator; heterogeneous endpoints and follow-up. | High methodological concerns |
| Harwani et al. DEN Open, 2025 [18] PMID: 40599977 | Hepatocellular carcinoma treated by EUS-RFA | Small case series | JBI Case Series | Only five patients; selected elderly/non-surgical population; no comparator; short follow-up; highly operator-dependent technique. | High methodological concerns |
| Goduguchinta et al. J Clin Med, 2025 [19] PMID: 40507720 | Various pancreatic neoplasms | Retrospective mixed-indication case series | JBI Case Series | Very small cohort; heterogeneous histologies and treatment intent; selected high-risk or surgery-refusing patients; no comparator; variable follow-up imaging. | High methodological concerns |

| Reference (PMID) | Indication target | Study design | Tool | Main methodological concerns | Overall judgment |
|---|---|---|---|---|---|
| Stouvenot et al. Endosc Int Open, 2025 [20] PMID: 40376026 | Pancreatic metastases from renal cell carcinoma | Small observational case series | JBI Case Series | Small sample; selected oligometa-static disease; no comparator; possi-ble overlap with prior Marseille RCC metastasis cohort; limited precision for adverse events. | High methodological concerns |
| Argentesi et al. Lancet, 2025 [21] PMID: 39929216 | Aldosterone-producing adrenal adenomas | Prospective proof-of-con-cept single-arm trial | JBI Case Series | No surgical or medical comparator; small proof-of-concept sample; short-term biochemical endpoints; highly selected left-sided lesions. | Moderate methodological concerns |
| Robles-Medranda et al. Gastrointest Endosc, 2024 [22] PMID: 38518978 | Unresectable pancreatic ductal adenocarcinoma | Single-center historic co-hort | JBI Case Series | No concurrent comparator; retro-spective/historic data capture with ambispective follow-up; survival confounded by stage, systemic ther-apy, and selection; limited sample. | High methodological concerns |
| Kongkam et al. Endosc Ultrasound, 2023 [23] PMID: 37969163 | Pancreatic cancer | Non-randomized open-label comparative pilot study | ROBINS-I | Small pilot sample; open-label de-sign; non-random allocation; incom-plete control of systemic therapy and prognostic variables; subjective pain/narcotic endpoints. | Serious risk of bias |
| Debraine et al. Clin Endo-crinol, 2024 [24] PMID: 37859570 | Benign pancreatic insu-linoma | Uncontrolled long-term observational series | JBI Case Series | No comparator; selected benign insu-linomas; limited sample; recurrence and repeat treatment may be incom-pletely captured across long follow-up. | Moderate methodological concerns |
| Cho et al. Gas-trointest En-dosc, 2023 [25] PMID: 37356635 | Left adrenal tumors | Prospective/observational single-arm series | JBI Case Series | Small sample; selected left-sided le-sions accessible by EUS; no compara-tor; response definitions heterogene-ous across adrenal pathologies. | High methodological concerns |

| Reference (PMID) | Indication target | Study design | Tool | Main methodological concerns | Overall judgment |
|---|---|---|---|---|---|
| Borrelli de Andreis et al. Pancreatology, 2023 [26] PMID: 37236853 | Pancreatic insulinoma | Single-center observational series | JBI Case Series | Small sample; no comparator; selected cases; procedural protocol and follow-up may not be fully standardized. | Moderate methodological concerns |
| Napoléon et al. Gastrointest Endosc, 2023 [27] PMID: 37059368 | Mixed pancreatic neoplasms / safety | Large retrospective multicenter safety cohort | JBI Case Series | Retrospective design; heterogeneous indications and techniques; no efficacy comparator; potential overlap with indication-specific European cohorts; adverse-event ascertainment may vary by center. | Moderate methodological concerns |
| Crinò et al. Clin Gastroenterol Hepatol, 2023 [28] PMID: 36871765 | Pancreatic insulinoma | Propensity-score matched non-randomized comparative study | ROBINS-I | Non-random allocation; residual confounding possible despite matching; patient selection and center expertise may influence outcomes; blinding not feasible. | Moderate risk of bias |
| Figueiredo Ferreira et al. Endosc Int Open, 2022 [30] PMID: 36262511 | Pancreatic/peripancreatic tumors and oligometastatic disease | Prospective multicenter observational single-arm study | JBI Case Series | Mixed lesions and treatment intent; no comparator; small indication-specific subgroups; heterogeneous endpoints and follow-up. | High methodological concerns |
| Thosani et al. Sci Rep, 2022 [31] PMID: 36192558 | Advanced pancreatic and periampullary adenocarcinoma | Prospective single-arm study | JBI Case Series | No comparator; advanced heterogeneous cancers; outcomes confounded by systemic therapy and natural history; limited sample size. | High methodological concerns |
| Younis et al. Eur J Gastroenterol Hepatol, 2022 [32] PMID: 36170679 | Premalignant pancreatic cystic neoplasms and neuroendocrine tumors | Small prospective single-arm study | JBI Case Series | Small sample; mixed indications; no comparator; radiological response endpoints; limited follow-up for recurrence/malignant progression. | High methodological concerns |

| Reference (PMID) | Indication target | Study design | Tool | Main methodological concerns | Overall judgment |
|---|---|---|---|---|---|
| Oh et al. Endosc Ultrasound, 2022 [33] PMID: 35083978 | Unresectable pancreatic cancer | Prospective observational single-arm study | JBI Case Series | No comparator; survival/local control confounded by disease stage and systemic therapy; small sample; short follow-up. | High methodological concerns |
| Marx et al. Dig Endosc, 2022 [34] PMID: 34963025 | Non-functioning pancreatic neuroendocrine tumors | Bicentric retrospective case series | JBI Case Series | Retrospective design; no comparator; selected small G1 lesions; limited follow-up; possible center overlap with related Marseille reports. | Moderate methodological concerns |
| Marx et al. Gastrointest Endosc, 2022 [35] PMID: 34902374 | Pancreatic insulinoma | Two-center retrospective case series | JBI Case Series | Small sample; no comparator; selected referral cases; follow-up and retreatment criteria may be heterogeneous; possible overlap with center-specific cohorts. | Moderate methodological concerns |
| Chanez et al. Cancers, 2021 [36] PMID: 34771431 | Pancreatic metastases from renal cell carcinoma | Prospective single-arm study | JBI Case Series | Small sample; selected oligometastatic RCC; no comparator; focal-control endpoint; systemic disease course and concurrent therapies may confound interpretation. | Moderate methodological concerns |
| Barthet et al. Endosc Int Open, 2021 [37] PMID: 34447860 | Pancreatic neuroendocrine tumors and pancreatic cystic neoplasms | Prospective long-term follow-up single-arm study | JBI Case Series | Mixed indications; no comparator; possible overlap with earlier Barthet cohort; small subgroups; response definitions differ by lesion type. | Moderate methodological concerns |
| Wang et al. Ann Palliat Med, 2021 [38] PMID: 33440967 | Unresectable pancreatic cancer | Preliminary single-arm study | JBI Case Series | Small preliminary cohort; no comparator; low-power protocol; survival and pain outcomes confounded by systemic therapy and disease progression. | High methodological concerns |
| de Nucci et al. Endosc Int Open, 2020 [39] PMID: 33269307 | Pancreatic neuroendocrine tumors | Prospective case series | JBI Case Series | Small sample; no comparator; selected lesions; limited long-term follow-up; response criteria not standardized across all cases. | High methodological concerns |

| Reference (PMID) | Indication target | Study design | Tool | Main methodological concerns | Overall judgment |
|---|---|---|---|---|---|
| Oh et al. Endoscopy, 2021 [40] PMID: 33063298 | Pancreatic microcystic serous cystic neoplasms | Retrospective single-arm study | JBI Case Series | Retrospective design; no comparator; benign disease with radiological response endpoint; small sample; limited clinical endpoint relevance. | Moderate methodological concerns |
| Barthet et al. Endoscopy, 2019 [41] PMID: 30669161 | Pancreatic neuroendocrine tumors and pancreatic cystic neoplasms | Prospective multicenter single-arm study | JBI Case Series | No comparator; mixed indications; small subgroups; possible overlap with later long-term Barthet/Barras reports; heterogeneous outcome definitions. | Moderate methodological concerns |
| Bang et al. Gastrointest Endosc, 2019 [42] PMID: 30120957 | Celiac ganglion RFA for pancreatic cancer pain palliation | Randomized controlled trial | RoB 2 | Subjective pain outcomes; blinding likely limited; small sample; indication is pain palliation rather than tumor ablation. | Some concerns |
| Choi et al. Endoscopy, 2018 [43] PMID: 29727904 | Benign solid pancreatic tumors | Prospective single-arm study | JBI Case Series | Small sample; no comparator; mixed benign solid lesions; selected patients; limited follow-up for recurrence. | Moderate methodological concerns |
| Crinò et al. J Gastrointestin Liver Dis, 2018 [44] PMID: 29557417 | Solid pancreatic neoplasms | Early feasibility case series | JBI Case Series | Very small early experience; no comparator; short follow-up; mainly technical endpoints; heterogeneous lesions. | High methodological concerns |
| Lakhtakia et al. Gastrointest Endosc, 2016 [45] PMID: 26394384 | Pancreatic insulinoma | Early small case series | JBI Case Series | Very small early human experience; no comparator; short/limited follow-up; selected patients; outcomes mainly symptom response. | High methodological concerns |
| Song et al. Gastrointest Endosc, 2016 [46] PMID: 26344883 | Unresectable pancreatic cancer | Initial clinical experience case series | JBI Case Series | Very small initial experience; no comparator; short follow-up; outcomes confounded by disease stage and concomitant therapy. | High methodological concerns |

Faraoni et al. Cancer Immunol Res, 2023 [29] PMID: 36367967 was not included since no clinical evidence was provided. **Abbreviations:** BD-IPMN, branch-duct intraductal papillary mucinous neoplasm; EUS-RFA, endoscopic ultrasound-guided radiofrequency ablation; JBI, Joanna Briggs Institute; PanNET, pancreatic neuroendocrine tumor; RCC, renal cell carcinoma; RoB 2, revised Cochrane risk-of-bias tool for randomized trials; ROBINS-I, Risk Of Bias In Non-randomized Studies of Interventions; WF/HRS, worrisome features/high-risk stigmata.

**Footnote:** For JBI-assessed uncontrolled studies, the table reports overall methodological concerns rather than a numerical score. For ROBINS-I, the reported judgment follows the usual categories of low, moderate, serious, critical risk of bias, or no information. The three non-assessed records are retained here only to document how records from the original evidence table should be handled in the revised manuscript.

**Supplementary Table S3**. Certainty of evidence according to GRADE classification

| Indication | Key outcomes | Inconsistency | Indirectness | Imprecision | Publication bias | Overall certainty |
|---|---|---|---|---|---|---|
| Pancreatic insulinoma | Symptom resolution; biochemical control of hypoglycemia; adverse events; recurrence/need for retreatment; comparison with surgery when available. | Not serious to serious: direction of effect generally consistent for short-term symptom control, but AE rates and retreatment requirements vary. | Not serious to serious: population and outcome are clinically relevant, but treatment intent is biochemical/symptomatic rather than oncologic cure; many patients were selected because surgery was unsuitable or refused. | Serious: small cohorts, few comparative data, wide confidence intervals for comparative endpoints, limited long-term recurrence estimates. | Suspected: small-study effects and preferential publication of technically successful experiences are possible. | LOW for short-term clinical/biochemical success and comparative effectiveness versus surgery; VERY LOW for long-term recurrence-free control. |
| Non-functioning pancreatic neuroendocrine neoplasms | Technical success; radiological response; complete ablation/loss of enhancement; adverse events; recurrence/progression; need for surgery. | Serious: response rates vary by lesion size, imaging modality, ablation protocol, and definition of complete response. | Serious: radiological response is a surrogate for oncologic adequacy; long-term disease-specific outcomes are rarely reported. | Serious: limited sample size, few events, short-to-intermediate follow-up in most studies. | Strongly suspected: small case series and positive early experiences likely over-represented. | VERY LOW to LOW for short-term radiological response; VERY LOW for durable oncologic control. |

| Indication | Key outcomes | Inconsistency | Indirectness | Imprecision | Publication bias | Overall certainty |
|---|---|---|---|---|---|---|
| Branch-duct IPMN and pancreatic cystic neoplasms | Technical success; cyst-volume reduction; disappearance of mural nodules/worrisome features; molecular response when available; pancreatitis/adverse events; local control; progression to malignancy. | Serious: studies differ in cyst type, size, worrisome features, ablation platform, number of sessions, and follow-up duration. | Serious: cyst shrinkage and disappearance of enhancement/mutations are surrogate endpoints; prevention of cancer remains unproven. | Serious: few studies, limited patient numbers, rare malignant transformation events. | Suspected: positive technical and radiological responses are more likely to be published. | VERY LOW to LOW for short-term cyst/radiological response; VERY LOW for cancer-prevention or long-term oncologic benefit. |
| Pancreatic ductal adenocarcinoma | Technical feasibility; adverse events; tumor necrosis/local control; pain or performance-status improvement; overall survival; downstaging. | Serious: variation in disease stage, tumor size, RFA route/settings, chemotherapy regimens, and endpoints. | Serious: local necrosis and imaging changes do not establish survival benefit; palliation, local control and survival are difficult to separate. | Serious: small cohorts, few comparative datasets, limited power for survival outcomes. | Strongly suspected: exploratory positive single-center experiences likely over-represented. | VERY LOW for survival, downstaging and oncologic benefit; LOW to VERY LOW for technical feasibility/safety in expert centers. |
| Pancreatic metastases from renal cell carcinoma | Technical success; focal control; radiological response; adverse events; avoidance or delay of systemic therapy/surgery; progression. | Serious: limited number of studies and heterogeneous lesion number, size, prior therapy, and systemic disease status. | Serious: focal control may not translate into systemic disease benefit or survival advantage. | Serious: very small sample sizes and limited event numbers. | Suspected: rare indication with publication likely driven by feasibility and positive experience. | VERY LOW for focal control and clinical benefit; LOW to VERY LOW for feasibility in selected expert-center patients. |

| Indication | Key outcomes | Inconsistency | Indirectness | Imprecision | Publication bias | Overall certainty |
|---|---|---|---|---|---|---|
| Adrenal gland adenoma | Technical success; feasibility of left adrenal access; biochemical cure or improvement; blood-pressure response; reduction in antihypertensive medication; radiological response; adverse events; need for adrenalectomy or repeat ablation. | Serious: available studies are very limited and differ in lesion selection, functional status, biochemical endpoints, follow-up duration, and definitions of clinical or biochemical response. | Serious: evidence mainly concerns highly selected left-sided adrenal lesions accessible from the stomach; findings may not be generalizable to right-sided adrenal lesions, non-functioning adrenal tumors, larger lesions, or patients eligible for standard adrenalectomy. | Serious: very small sample sizes, few events, limited follow-up, and absence of adequately powered comparative data against adrenalectomy or optimized medical therapy. | Suspected: early feasibility and proof-of-concept experiences with favorable technical or biochemical outcomes are more likely to be reported than unsuccessful or complicated procedures. | VERY LOW for durable biochemical cure, blood-pressure control, and comparison with adrenalectomy or medical therapy; LOW to VERY LOW for technical feasibility and short-term safety in selected expert-center patients. |
| Hepatocellular carcinoma and other extra-pancreatic tumors | Technical success; feasibility of EUS-guided access; radiological response; local tumor control; adverse events; need for additional local or systemic therapy; progression-free survival; overall survival. | Serious: evidence is limited to very small and heterogeneous reports, with variation in target organ, tumor biology, lesion size, previous treatments, ablation technique, and follow-up assessment. | Very serious: local radiological response is a surrogate endpoint and may not translate into durable oncological control, progression-free survival, or survival benefit; results cannot be generalized across different extra-pancreatic tumor types or compared directly with established percutaneous, surgical, or transarterial therapies. | Very serious: extremely small patient numbers, sparse outcome events, no comparative data, and insufficient power to assess uncommon adverse events or long-term oncological outcomes. | Strongly suspected: rare and technically novel applications are likely subject to selective publication of successful cases and under-reporting of negative or complicated experiences. | VERY LOW for local control, survival benefit, and clinical utility; LOW to VERY LOW for technical feasibility and short-term safety in highly selected lesions managed in expert centers. |

| Indication | Key outcomes | Inconsistency | Indirectness | Imprecision | Publication bias | Overall certainty |
|---|---|---|---|---|---|---|
| Celiac ganglion RFA | Pain reduction; change in opioid consumption; quality of life or performance status when available; technical success; adverse events; need for additional pain interventions; duration of analgesic response. | Not serious to serious: the main evidence is derived from a randomized comparison, but outcome reporting is limited and pain response may vary according to baseline pain severity, disease stage, opioid use, and concomitant oncological or supportive treatments. | Serious: this indication concerns pain palliation rather than tumor ablation; therefore, the findings are not applicable to the oncological efficacy of EUS-RFA for pancreatic cancer or other tumors. Pain scores and opioid use are clinically relevant but subjective and influenced by multiple non-procedural factors. | Serious: evidence is based on a limited number of patients and short-term follow-up; estimates for durability of analgesia, quality-of-life benefit, and uncommon adverse events remain imprecise. | Possible: although randomized evidence reduces some bias, publication bias cannot be excluded because few studies have evaluated this indication and positive analgesic results may be more likely to be reported. | * LOW for short-term pain palliation in pancreatic cancer; VERY LOW for durability of analgesic benefit and impact on quality of life. |

**Abbreviations:** AE, adverse event; BD-IPMN, branch-duct intraductal papillary mucinous neoplasm; EUS-RFA, endoscopic ultrasound-guided radiofrequency ablation; GRADE, Grading of Recommendations Assessment, Development and Evaluation; NF, non-functioning; PCN, pancreatic cystic neoplasm; PDAC, pancreatic ductal adenocarcinoma; RCC, renal cell carcinoma. *For celiac ganglion RFA, certainty was assessed only for pain-palliation outcomes and not for tumor-ablation efficacy. Extra-pancreatic tumor-ablation indications were graded separately because their biological behavior, treatment intent, comparator standards, and clinically meaningful endpoints differ substantially from pancreatic tumor-ablation indications.

**Supplementary Table S4**. Handling of potential study overlap

| Potential overlap group | Reports concerned | Risk of double-counting |
|---|---|---|
| Marseille/Barthet-Barras pancreatic cystic neoplasm/IPMN reports | Barthet et al., Endoscopy 2019, PMID 30669161; Barthet et al., Endosc Int Open 2021, PMID 34447860; Barras et al., Gastrointest Endosc 2026, PMID 41903818. | High for pancreatic cystic neoplasm/IPMN patients and lesions if the reports are summed as independent cohorts. Lower for NET-specific data when extracted separately. |
| Marx 2022 pancreatic NET reports | Marx et al., Dig Endosc 2022, PMID 34963025; Marx et al., Gastrointest Endosc 2022, PMID 34902374. | Low to moderate. Patient overlap is unlikely because functional insulinoma and non-functioning PanNET cohorts were reported as different disease entities, but center-level overlap exists. |
| Chulalongkorn/Kongkam PDAC reports | Kongkam et al., Endosc Ultrasound 2023, PMID 37969163; Kongkam et al., Gastrointest Endosc 2025, PMID 40680897. | Moderate to high if survival outcomes and patient numbers are pooled across both reports, because partial overlap cannot be excluded from the available report-level information. |

| Potential overlap group | Reports concerned | Risk of double-counting |
|---|---|---|
| French/Marseille RCC pancreatic metastasis reports | Chanez et al., Cancers 2021, PMID 34771431; Stouvenot et al., Endosc Int Open 2025, PMID 40376026. | Moderate. Exact overlap cannot be excluded without individual patient-level data, especially because both reports concern a rare indication in high-volume French centers. |
| French RAFPAN safety registry versus French/European indication-specific series | Napoléon et al., Gastrointest Endosc 2023, RAFPAN study, PMID 37059368; potentially overlapping French/European reports including Barthet 2019/2021, Chanez 2021, Marx 2022, Debraine 2024, Rizzatti 2025, and other high-volume-center experiences. | High for safety denominators if RAFPAN is pooled with individual French/European series. Lower for qualitative risk-factor interpretation because the study objective is safety rather than indication-specific efficacy. |
| Rizzatti 2025 prospective PanNET cohort and earlier European PanNET reports | Rizzatti et al., Endosc Int Open 2025, PMID 41142260; earlier European PanNET reports including Barthet 2019/2021 and Marx 2022 reports. | Moderate if PanNET patient counts from Rizzatti 2025 are summed together with prior European reports without considering possible center-level or period overlap. |
| Italian/European insulinoma reports and comparative insulinoma study | Crino et al., Clin Gastroenterol Hepatol 2023, PMID 36871765; Borrelli de Andreis et al., Pancreatology 2023, PMID 37236853; other small insulinoma series from high-volume centers. | Moderate. Exact overlap cannot be excluded for some single-center insulinoma experiences, particularly when study periods and centers overlap with multicenter comparative datasets. |
| Mixed pancreatic neoplasm/safety cohorts versus indication-specific reports | Crino et al. 2018, Goduguchinta et al. 2025, Okasha et al. 2025, Napoléon/RAFPAN 2023, and other mixed-indication reports. | Moderate for global patient/procedure totals and adverse-event denominators; lower for qualitative feasibility description. |

**Abbreviations:** BD-IPMN, branch-duct intraductal papillary mucinous neoplasm; EUS-RFA, endoscopic ultrasound-guided radiofrequency ablation; PanNET, pancreatic neuroendocrine tumor; PDAC, pancreatic ductal adenocarcinoma; RCC, renal cell carcinoma.

**Supplementary Table S5**. EUS-RFA technique and study designs

| Author/year | Indication | Patients/lesions/procedures | Lesion size | Device | Power settings | Antibiotic prophylaxis | Pancreatitis prophylaxis | Follow-up | Response definition |
|---|---|---|---|---|---|---|---|---|---|
| Barras et al., 2026 | BD-IPMN with WF/HRS | 50 patients; 58 lesions; 62 procedures | NR; smaller cyst size associated with better outcomes | NR | NR | NR | NR | Mean 4.1 +/- 2.8 years after RFA; long-term observation from IPMN diagnosis | Local control: resolution of WF/HRS with no cancer arising from treated lesion; radiologic response as disappearance or size decrease |
| Platt et al., 2026 | Localized non-functioning panNETs | 17 patients; 18 lesions | Mean 1.4 +/- 0.5 cm pre-ablation | NR | NR | NR | NR | CT/MRI/68Ga-DOTATATE PET; mean clinical follow-up 650 days | Complete response: loss of enhancement and SUVmax; partial response: decrease in size, enhancement, or SUVmax |
| Krishna et al., 2026 | Large BD-IPMN | 25 participants; 30 BD-IPMNs; 41 sessions | Mean diameter 4.6 +/- 1.7 cm | 19G EUS-RFA needle | Application until maximum 45 s or 400-ohm impedance threshold | NR | NR | Mean follow-up 18 +/- 5 months; cyst imaging and cyst-fluid molecular follow-up | >=50% cyst-volume reduction; complete response defined as >=90% volume reduction; exploratory KRAS/GNAS molecular response |
| Ardengh et al., 2025 | Small functioning and non-functioning PNETs | 4 patients/lesions; 5 sessions | All <15 mm | NR | NR | NR | NR | MRI immediately or 30 days after RFA | MRI-based loss of tumor enhancement / complete nodule destruction |
| Rizzatti et al., 2025 | Functional and non-function- | 60 patients; 30 insulinomas and 30 | Mean 14.5 +/- 4.5 mm | EUSRA 19G needle | 30 W for lesions <10 mm; 50 W for le- | Antibiotics according | Rectal indomethacin or | Follow-up for 1 year; clinical contacts/visits at days 7, 15, 30, | Insulinoma: disappearance of hormonal syndrome; |

| Author/year | Indication | Patients/lesions/procedures | Lesion size | Device | Power settings | Antibiotic prophylaxis | Pancreatitis prophylaxis | Follow-up | Response definition |
|---|---|---|---|---|---|---|---|---|---|
| | ing PanNETs | NF-PanNETs | | | sions >10 mm; delivery stopped at impedance rise | to local protocol | diclofenac before procedure | 90 and every 3 months thereafter | NF-PanNET: absence of enhancing tissue and no detectable lesion on MRI/CT and Gallium-PET |
| Kovacevic et al., 2026 | Insulinoma | 17 patients; 23 procedures | <25 mm | Dedicated EUS-RFA needle | NR | NR | NR | Mean follow-up 15.5 months | Clinical success: symptom improvement with documented increase in blood glucose |
| Kongkam et al., 2025 | Unresectable PDAC <=4 cm | 12 EUS-RFA patients; 35 controls | <=4 cm | NR | NR | NR | NR | 1-year survival/local progression follow-up | Survival, local progression, and response rates after propensity matching / inverse probability weighting |
| Okasha et al., 2025 | Insulinoma and advanced pancreatic adenocarcinoma | 13 EUS-RFA patients | Mean masses 20.6 mm; insulinomas 17.4 mm | 19G EUSRA needle, Taewoong | NR | NR | NR | Mean follow-up 12.4 months | Insulinoma: complete clinical cure; adenocarcinoma: size decrease/downstaging assessment |
| Harwani et al., 2025 | Hepatocellular carcinoma | 5 patients | Lesions <3 cm | NR | NR | NR | Not applicable | Alpha-fetoprotein at 1 month; CT/triple-phase CT | Reduction or complete radiological response on CT; AFP reduction |
| Goduguchinta et al., 2025 | Mixed pancreatic neoplasms | 8 patients | Median 12.7 x 10.3 mm; range 11.0 x 8.5 | 19G RFA needle, | 10 W for <15 mm solid tumors; 20 W for >15 mm | NR | NR | CT abdomen/pelvis every 3-6 months | Tumor-size reduction / radiological response |

| Author/year | Indication | Patients/lesions/procedures | Lesion size | Device | Power settings | Antibiotic prophylaxis | Pancreatitis prophylaxis | Follow-up | Response definition |
|---|---|---|---|---|---|---|---|---|---|
| | | | to 25.0 x 20.0 mm for solid lesions | Taewoong | lesions; 50 W for cystic lesions with mural nodules | | | | |
| Stouvenot et al., 2025 | Pancreatic RCC metastases | 8 patients; 11 lesions | Mean 13.9 +/- 3.9 mm | 18G or 19G dedicated RFA needles | NR | NR | NR | Contrast-enhanced CT at 2-5 months, 1 year, and end of follow-up | Complete response: necrosis with no contrast enhancement or lesion disappearance; partial response otherwise as reported |
| Argentesi et al., 2025 | Left aldosterone-producing adrenal adenoma | 28 participants; 35 ablations | NR | 19G ablation catheter | Incremental 10-20 s treatments/burns | NR | Not applicable | Molecular imaging at 3 months; biochemical/clinical assessment at 6 months | Reduction/disappearance of tracer uptake; biochemical and clinical cure of primary aldosteronism/hypertension |
| Robles-Medranda et al., 2024 | Unresectable PDAC | 26 patients | Median 39.5 mm before RFA; 26 mm at 6 months among survivors | 19G RFA needle electrode | 50 W | Ceftriaxone 1 g IV before procedure | NR | CT at 1 month to assess need for repeat ablation; 6-month clinical/imaging follow-up | Feasibility/safety; performance status; local control by tumor size and necrotic area; overall survival |
| Kongkam et al., 2023 | Unresectable PDAC; EUS-RFA + chemotherapy | 14 EUS-RFA + chemotherapy; 14 chemotherapy controls; 30 | Mean maximal diameter 62.2 +/- 21.0 mm in | 19G RFA needle | 50 W | NR | Yes, according to extracted review table | 6-month assessment | Tumor necrosis, tumor diameter, narcotic dose, and 6-month mortality |

| Author/year | Indication | Patients/lesions/procedures | Lesion size | Device | Power settings | Antibiotic prophylaxis | Pancreatitis prophylaxis | Follow-up | Response definition |
|---|---|---|---|---|---|---|---|---|---|
| | | EUS-RFA procedures | RFA group | | | | | | |
| Debraine et al., 2024 | Benign insulinoma | 11 patients | Mean 11 mm | NR | NR | NR | NR | Clinical/biochemical/imaging follow-up at 3, 6, 12 months and annually | Clinical and biological remission; radiological resolution or residual tumor |
| Cho et al., 2023 | Left adrenal tumors | 11 patients | NR | NR | NR | NR | Not applicable | Follow-up not specified in abstract | Complete or partial response |
| Borrelli de Andreis et al., 2023 | Insulinoma | 10 patients | Mean 11.9 +/- 3.3 mm | NR | NR | NR | NR | Radiological response within 3 months; median follow-up 19.5 months | Symptom remission with normal blood glucose; intralesional necrosis / complete radiologic response |
| Napoleon et al., 2023 (RAFPAN) | Mixed pancreatic neoplasms / safety registry | 100 patients; 104 neoplasms | Size <20 mm independently associated with complete ablation; detailed size NR | NR | NR | NR | NR | NR | Complete response, partial response, no response; adverse events and risk factors |
| Crino et al., 2023 | Insulinoma; EUS-RFA vs surgery | 89 EUS-RFA patients after matching; 89 surgery controls | Matched by lesion size; detailed size NR in abstract | NR | NR | NR | NR | Median follow-up 23 months for EUS-RFA | Clinical efficacy, adverse events, hospital stay, recurrence |
| Faraoni et al., 2023 | Preclinical/translational pancreatic cancer | Not applicable | Not applicable | Not applicable | Not applicable | Not applicable | Not applicable | Not applicable | Preclinical/translational study; not a clinical procedural cohort |

| Author/year | Indication | Patients/lesions/procedures | Lesion size | Device | Power settings | Antibiotic prophylaxis | Pancreatitis prophylaxis | Follow-up | Response definition |
|---|---|---|---|---|---|---|---|---|---|
| Ferreira et al., 2022 | Pancreatic/peri-pancreatic tumors and oligometastatic disease | 29 patients; 35 lesions | NR | NR | NR | NR | NR | 6- and 12-month follow-up | Radiological complete response, significant partial response (>50% decrease), or <50% decrease; insulinoma symptom correction |
| Thosani et al., 2022 | Advanced pancreatic/peri-ampullary adenocarcinoma | 10 patients; 22 sessions | NR | NR | NR | NR | NR | >30 months follow-up; imaging and survival follow-up | Tumor progression/regression on imaging; survival |
| Younis et al., 2022 | Premalignant PCNs and small PNETs | 12 patients; 5 PCNs and 7 PNETs | PCNs median 36 mm (12-60); PNETs median 8.9 mm (6-18) | 19G dedicated needle | NR | NR | NR | Median follow-up 7 months | Radiological complete response, partial response, or failure; insulinoma symptom resolution |
| Oh et al., 2022 | Unresectable pancreatic cancer | 22 patients; 107 sessions | NR | 19G RFA needle | 50 W | Yes, according to extracted review table | NR | Median follow-up 21.23 months | Overall survival and progression-free survival |
| Marx et al., 2022 (NF-pNET) | Non-functioning pNETs | 27 patients/lesions | Mean 14.0 +/- 4.6 mm | NR | NR | NR | NR | Mean follow-up 15.7 +/- 12.2 months | Complete treatment response on cross-sectional imaging / complete necrosis |
| Marx et al., 2022 (insulinoma) | Insulinoma | 7 patients | <2 cm | NR | NR | NR | NR | Median follow-up 21 months | Immediate hypoglycemia relief; com- |

| Author/year | Indication | Patients/lesions/procedures | Lesion size | Device | Power settings | Antibiotic prophylaxis | Pancreatitis prophylaxis | Follow-up | Response definition |
|---|---|---|---|---|---|---|---|---|---|
| | | | | | | | | | plete response on cross-sectional imaging |
| Chanez et al., 2021 | Pancreatic RCC metastases | 12 patients; 21 metastases; 26 procedures | Mean 17 mm (range 3-35) | 19G EUS-RFA needle electrode | 50 W; 30-60 s for lesions >20 mm | Cefotaxime | Rectal indomethacin | CT at 2 months after each procedure, then every 3-6 months | Complete response: disappearance or absence of arterial contrast uptake; partial/stable/progressive by contrast uptake and size |
| Barthet et al., 2021 | PanNETs and pancreatic cystic neoplasms | 29 patients; 14 NETs and 17 cystic tumors | NETs mean 13.4 mm (10-20); cystic tumors mean 29.1 mm (9-60) | RFA cooling needle | 50 W; impedance <500 ohm in extracted review table | Yes | Yes | Prospective 1-year assessment then annual follow-up; mean 42.9 months | NET complete disappearance; PCN significant response / mural-nodule resolution |
| Wang et al., 2021 | Unresectable pancreatic cancer | 11 patients | One reported lesion 29.7 mm; overall size NR | RITA 1500X RF generator | 5-10 W for 90 s | NR | NR | Imaging and CA19-9 at 1 month; follow-up 2-12 months | Lesion size, CA19-9, ADC value, and ablated area |
| de Nucci et al., 2020 | P-NETs | 10 patients; 11 lesions | Mean 14.5 mm (range 9-20) | NR | NR | NR | NR | CT at 6 and 12 months | Complete disappearance / radiological normalization |
| Oh et al., 2020 | Microcystic serous cystic neoplasms | 13 patients | Baseline median volume 37.82 mL | 19G RFA needle | NR | NR | NR | Median follow-up 9.21 months | Radiological partial response by cyst-volume decrease |
| Barthet et al., 2019 | PanNETs and pancreatic cystic neoplasms | 29 patients; 14 NETs and 17 cystic tumors | NETs mean 13.1 mm (10-20); PCNs | 18G RFA cooling needle | NR | Improved protocol included | Improved protocol included | 1-year follow-up | NET disappearance; PCN disappearance or >50% size |

| Author/year | Indication | Patients/lesions/procedures | Lesion size | Device | Power settings | Antibiotic prophylaxis | Pancreatitis prophylaxis | Follow-up | Response definition |
|---|---|---|---|---|---|---|---|---|---|
| | | | mean 28 mm (9-60) | | | prophylaxis; details not specified in abstract | prophylaxis; details not specified in abstract | | reduction; mural-nodule resolution |
| Bang et al., 2019 | Celiac ganglion RFA for pancreatic cancer pain | 12 EUS-RFA patients; 14 EUS-CPN controls | Not applicable | 1F monopolar probe passed through 19G FNA needle | NR | NR | Not applicable | 2 and 4 weeks | Pain severity by EORTC PAN26; QoL and opioid use |
| Choi et al., 2018 | Benign solid pancreatic tumors | 10 patients; 16 sessions | Median 20 mm (range 8-28) | RFA electrode | 50 W | NR | NR | Median follow-up 13 months | Radiological complete response |
| Crino et al., 2018 | Solid pancreatic neoplasms | 9 referred; 8 treated | NR | 18G internally cooled electrode | NR | NR | NR | Mean follow-up 6 months | Feasibility; ablated area within tumor; early/late adverse events |
| Lakhtakia et al., 2016 | Insulinoma | 3 patients | NR | Prototype 19G internally cooled needle electrode | 50 W | NR | NR | 11-12 months | Symptom relief and biochemical improvement |
| Song et al., 2016 | Unresectable pancreatic cancer | 6 patients | Median 3.8 cm (range 3-9) | 18G endoscopic RFA electrode | 20-50 W for 10 s | NR | NR | NR | Technical feasibility and safety |

Abbreviations: AE, adverse event; BD-IPMN, branch-duct intraductal papillary mucinous neoplasm; CT, computed tomography; EUS-CPN, EUS-guided celiac plexus neurolysis; EUS-RFA, endoscopic ultrasound-guided radiofrequency ablation; HCC, hepatocellular carcinoma; MRI, magnetic resonance imaging; NA, not applicable; NF, non-functioning; NR, not reported; PanNET/pNET/PNET, pancreatic neuroendocrine tumor; PCN, pancreatic cystic neoplasm; PDAC, pancreatic ductal adenocarcinoma; RCC, renal cell carcinoma; RFA, radiofrequency ablation; WF/HRS, worrisome features/high-risk stigmata.

**Supplementary Table S6**. Incidence of adverse events

| Reference | Indication | Overall incidence of adverse events | Incidence of post-RFA pancreatitis |
|---|---|---|---|
| Barras et al., 2026; PMID: 41903818 | BD-IPMN with worrisome features/high-risk stigmata | 27% of procedures | 3 patients; 3/50 patients (6.0%) or 3/62 procedures (4.8%) |
| Platt et al., 2026; PMID: 41656678 | Non-functioning PanNET | NR; one post-ablation pancreatitis reported | 1/17 patients (5.9%) |
| Krishna et al., 2026; PMID: 41584732 | Large BD-IPMN | 5/41 procedures (12.2%) | NR |
| Ardengh et al., 2025; PMID: 41467165 | Small pancreatic neuroendocrine tumors | 1/5 sessions (20.0%); mild duodenal bleeding | 0 reported |
| Rizzatti et al., 2025; PMID: 41142260 | Functional and non-functioning PanNETs | 9/60 patients (15.0%); insulinoma 4/30 (13.3%); NF-PanNET 5/30 (16.7%) | NR |
| Kovacevic et al., 2026; PMID: 41120112 | Insulinoma | 4/23 procedures (17.4%) | NR |
| Kongkam et al., 2025; PMID: 40680897 | Unresectable PDAC <=4 cm | Mild abdominal pain in 10% of RFA procedures | 0 reported |
| Okasha et al., 2025; PMID: 40620311 | Insulinoma and pancreatic adenocarcinoma | 0/13 EUS-RFA patients; no minor or major complications in EUS-RFA group | 0/13 EUS-RFA patients |
| Harwani et al., 2025; PMID: 40599977 | Hepatocellular carcinoma | 0/5 patients; no procedural adverse events | Not applicable |
| Goduguchinta et al., 2025; PMID: 40507720 | Mixed pancreatic neoplasms | 3/8 patients (37.5%) | NR; pancreatitis included among reported complications |
| Stouvenot et al., 2025; PMID: 40376026 | Pancreatic metastases from renal cell carcinoma | 3/8 patients (37.5%) | 1/8 patients (12.5%) |
| Argentesi et al., 2025; PMID: 39929216 | Aldosterone-producing adrenal adenoma | No major early safety hazards reported | Not applicable |
| Robles-Medranda et al., 2024; PMID: 38518978 | Unresectable/metastatic PDAC | No major adverse events reported | 0 reported |

| Reference | Indication | Overall incidence of adverse events | Incidence of post-RFA pancreatitis |
|---|---|---|---|
| Kongkam et al., 2023; PMID: 37969163 | PDAC; EUS-RFA plus chemotherapy | NR in abstract | NR |
| Debraine et al., 2024; PMID: 37859570 | Benign insulinoma | No notable complications reported | 0 reported |
| Cho et al., 2023; PMID: 37356635 | Left adrenal tumors | 5/11 patients (45.5%); self-limiting mild abdominal pain only | Not applicable |
| Borrelli de Andreis et al., 2023; PMID: 37236853 | Insulinoma | 2/10 patients (20.0%); mild abdominal pain | 0 reported |
| Napoleon et al., 2023; PMID: 37059368 | Mixed pancreatic neoplasms; RAFPAN safety registry | 22 AEs/100 patients (22.0%) | NR |
| Crino et al., 2023; PMID: 36871765 | Insulinoma; EUS-RFA vs surgery | 18.0% after EUS-RFA | NR |
| Faraoni et al., 2023; PMID: 36367967 | Translational/preclinical pancreatic cancer study | Not applicable to clinical safety table | Not applicable |
| Figueiredo Ferreira et al., 2022; PMID: 36262511 | Pancreatic/peripancreatic tumors and oligometastatic disease | 12/29 patients (41.4%); 59% had no collateral effects | 3/29 patients (10.3%) |
| Thosani et al., 2022; PMID: 36192558 | Advanced pancreatic/periampullary adenocarcinoma | NR in abstract | NR |
| Younis et al., 2022; PMID: 36170679 | PCN and small PNETs | 3/12 patients (25.0%) | 1/12 patients (8.3%) |
| Oh et al., 2022; PMID: 35083978 | Unresectable pancreatic cancer | 4/107 sessions (3.74%) | NR |
| Marx et al., 2022; PMID: 34963025 | Non-functioning pNETs | 4/27 patients (14.8%); all reported as periprocedural acute pancreatitis | 4/27 patients (14.8%) |
| Marx et al., 2022; PMID: 34902374 | Insulinoma | 3/7 patients (42.9%); minor AEs; one fatal delayed collection also reported | NR |
| Chanez et al., 2021; PMID: 34771431 | Pancreatic metastases from renal cell carcinoma | 2 severe complications/12 patients (16.7%) | NR |
| Barthet et al., 2021; PMID: 34447860 | PanNETs and pancreatic cystic neoplasms; long-term follow-up | No other long-term side effects reported | 0 reported during long-term follow-up |
| Wang et al., 2021; PMID: 33440967 | Unresectable pancreatic cancer | No major adverse events reported | 0 reported |

| Reference | Indication | Overall incidence of adverse events | Incidence of post-RFA pancreatitis |
|---|---|---|---|
| de Nucci et al., 2020; PMID: 33269307 | Pancreatic neuroendocrine tumors | 2/10 patients (20.0%); mild abdominal pain | 0 reported |
| Oh et al., 2021; PMID: 33063298 | Pancreatic microcystic serous cystic neoplasms | 1/13 patients (7.7%); self-limited abdominal pain | 0 reported |
| Barthet et al., 2019; PMID: 30669161 | PanNETs and pancreatic cystic neoplasms | 3/29 patients (10.3%) | 1/29 patients (3.4%) |
| Bang et al., 2019; PMID: 30120957 | Celiac ganglion RFA for pancreatic cancer pain palliation | NR in abstract; pain-palliation indication, not tumor ablation | Not applicable |
| Choi et al., 2018; PMID: 29727904 | Benign solid pancreatic tumors | 2/10 patients (20.0%) or 2/16 sessions (12.5%) | NR |
| Crino et al., 2018; PMID: 29557417 | Solid pancreatic neoplasms | No early or late major adverse events; 3 mild abdominal pain cases | 0 reported |
| Lakhtakia et al., 2016; PMID: 26394384 | Insulinoma | 0/3 patients (0%) | 0/3 patients (0%) |
| Song et al., 2016; PMID: 26344883 | Unresectable pancreatic cancer | 2/6 patients (33.3%); mild abdominal pain only | 0/6 patients (0%) |

**Abbreviations:** AE, adverse event; BD-IPMN, branch-duct intraductal papillary mucinous neoplasm; EUS-RFA, endoscopic ultrasound-guided radiofrequency ablation; NR, not reported in the abstract/source material available for extraction; PanNET, pancreatic neuroendocrine tumor; PCN, pancreatic cystic neoplasm; PDAC, pancreatic ductal adenocarcinoma. Denominators are reported as provided by each study; rates were not pooled because adverse events were variably reported per patient, per lesion, per procedure, or per treatment session. For secondary evidence syntheses, isolated case reports, and preclinical/translational studies, clinical study-level safety extraction was considered not applicable.
